# Supplementary material for: Systematic identification of needlefish (Belonidae) species using molecular genetic and morphological markers in the Mediterranean and Black Seas
Source: PLoS One. 2025 Feb 28;20(2):e0315401. doi: 10.1371/journal.pone.0315401 (PMC11870352; doi:10.1371/journal.pone.0315401)
Supplement: S1 Table — (PDF) [file pone.0315401.s001.pdf]

**Systematic Identification of Needlefish (Belonidae) Species using Molecular Genetic and Morphological Markers in the Mediterranean and Black Seas**

**DOI:** 10.5281zenodo.14249800

**Link:** https://zenodo.org/records/14249800

**S1 Table:** The sequences used in the study from NCBI and BOLD databases.

| Gene Region | Species | Country | NCBI | BOLD | Region |
|---|---|---|---|---|---|
| COI | *Belone belone* | Turkiye | OK148139.1 | | Black Sea |
| | *Belone belone* | Turkiye | KY176405.1 | | Marmara Sea |
| | *Belone belone* | Germany | KJ204731.1 | | North Sea |
| | *Belone belone* | France | JN231237.1 | | Celtik Sea |
| | *Belone belone* | Turkiye | KC500332.1 | | Black Sea |
| | *Belone belone* | Portugal | JQ774572.1 | | Atlantıic Ocean |
| | *Belone belone* | Turkiye | | ERDK003-21 | Marmara Sea |
| | *Belone belone* | Sweden | KJ128428.1 | | North Sea |
| | *Belone belone* | Norway | | NOFIS046-10 | North Sea |
| | *Belone belone* | Norway | | NOFIS047-10 | North Sea |
| | *Belone belone* | Israel | | BIM881-21 | Mediterranean Sea |
| | *Belone belone* | Turkiye | MZ723128.1 | | Marmara Sea |
| | *Belone belone* | Turkiye | | | Aegean Sea |
| | *Belone svetovidovi* | Turkiye | OQ329419.1 | | Marmara Sea |
| | *Belone svetovidovi* | Turkiye | OQ329422.1 | | Marmara Sea |
| | *Belone svetovidovi* | Turkiye | OQ329420.1 | | Marmara Sea |
| | *Belone svetovidovi* | Turkiye | OQ329423.1 | | Marmara Sea |
| | *Belone svetovidovi* | Turkiye | OQ329421.1 | | Marmara Sea |
| | *Belone svetovidovi* | Turkiye | OR234700.1 | | Black Sea |
| | *Belone svetovidovi* | Turkiye | OR234699.1 | | Black Sea |
| | *Belone svetovidovi* | Turkiye | OR234698.1 | | Black Sea |
| | *Belone svetovidovi* | Turkiye | OR234697.1 | | Black Sea |
| | *Belone svetovidovi* | Turkiye | OR234696.1 | | Black Sea |
| | *Belone svetovidovi* | Turkiye | OR234695.1 | | Marmara Sea |
| | *Belone svetovidovi* | Turkiye | OR234694.1 | | Marmara Sea |
| | *Belone svetovidovi* | Turkiye | OR234693.1 | | Marmara Sea |
| | *Belone svetovidovi* | Turkiye | OR234691.1 | | Marmara Sea |
| | *Belone svetovidovi* | Turkiye | OR234692.1 | | Marmara Sea |
| | *Tylosurus acus imperialis* | Turkiye | KY176687.1 | | Mediterranean Sea |
| | *Tylosurus acus* | Indian | MW709397.1 | | Indian Ocean |
| | *Tylosurus acus* | Bali | MH085783.1 | | Java Sea |
| | *Tylosurus acus* | Bali | MH085782.1 | | Java Sea |
| | *Tylosurus acus* | Mozambique | JF494755.1 | | Indian Ocean |
| | *Tylosurus acus* | Philippines | KC970513.1 | | Philippine Sea |
| | *Tylosurus acus melanotus* | China | KU605633.1 | | South China Sea |
| | *Tylosurus acus melanotus* | Indian | MH377844.1 | | Indian Ocean |
| | *Tylosurus acus melanotus* | Indian | MH377843.1 | | Indian Ocean |
| | *Tylosurus acus melanotus* | Indian | MH377842.1 | | Indian Ocean |

| | | | | | |
|---|---|---|---|---|---|
| | *Tylosurus acus melanotus* | Taiwan | KU943252.1 | | Philippine Sea |
| | *Tylosurus acus acus* | Brazil | GU702391.1 | | Atlantıic Ocean |
| | *Tylosurus acus acus* | Brazil | GU702389.1 | | Atlantıic Ocean |
| | *Tylosurus acus acus* | Brazil | GU702387.1 | | Atlantıic Ocean |
| | *Tylosurus crocodilus* | Vietnam | MK777132.1 | | South China Sea |
| | *Tylosurus crocodilus* | India | MT265055.1 | | Arabian Sea |
| | *Tylosurus crocodilus* | Philippines | OR113810.1 | | Philippine Sea |
| | *Tylosurus crocodilus* | Tanzania | ON644992.1 | | Indian Ocean |
| | *Tylosurus crocodilus* | India | KX433138.1 | | Arabian Sea |
| | *Tylosurus crocodilus* | China | OQ552922.1 | | South China Sea |
| | *Tylosurus gavialoides* | Australia | | AMS101-08 | Indian Ocean |
| | *Tylosurus gavialoides* | Philippines | KF715036.1 | | Philippine Sea |
| | *Tylosurus gavialoides* | Philippines | KF715035.1 | | Philippine Sea |
| | *Ablennes hians* | USA | MT323766.1 | | Atlantic Ocean |
| **12s rRNA** | *Tylosurus acus imperialis* | Israel | AF231572.1 | | Mediterranean Sea |
| | *Tylosurus acus acus* | Bermuda | *AF231571.1* | | Atlantic Ocean |
| | *Tylosurus acus pacificus* | *Panama* | *AF231575.1* | | Pasific Ocean |
| | *Tylosurus acus rafale* | *Senegal* | *AF231576.1* | | Atlantic Ocean |
| | *Tylosurus acus melanotus* | *Panama* | *AF231574.1* | | Pasific Ocean |
| | *Tylosurus acus melanotus* | *Philippines* | *AF231573.1* | | Pasific Ocean |
| | *Tylosurus crocodilus crocodilus* | *Qatar* | *MH248221.1* | | Arabian Sea |
| | *Tylosurus crocodilus* | *Panama* | *AF231578.1* | | Atlantic Ocean |
| | *Tylosurus crocodilus crocodilus* | *Tonga* | *AF231579.1* | | Pasific Ocean |
| | *Belone belone* | *France* | *AY141340.1* | | Mediterranean Sea |
| | *Ablennes hians* | *Philippines* | *AF231540.1* | | Pasific Ocean |
