# Supplementary material for: Systematic identification of needlefish (Belonidae) species using molecular genetic and morphological markers in the Mediterranean and Black Seas
Source: PLoS One. 2025 Feb 28;20(2):e0315401. doi: 10.1371/journal.pone.0315401 (PMC11870352; doi:10.1371/journal.pone.0315401)
Supplement: Attachment — Submitted filename: PONE-D-24-33951_reviewer.pdf [file pone.0315401.s004.pdf]

PLOS ONE

Systematic Identification of Needle Fish (Belonidae) Species using Molecular Genetic and Morphological Markers in the Mediterranean Sea

--Manuscript Draft--

Manuscript Number:	PONE-D-24-33951
Article Type:	Research Article
Full Title:	Systematic Identification of Needle Fish (Belonidae) Species using Molecular Genetic and Morphological Markers in the Mediterranean Sea
Short Title:	Systematic Identification of Needle Fish in the Mediterranean Sea
Corresponding Author:	Cemal Turan, PhD<br>Iskenderun Technical University: Iskenderun Teknik Universitesi<br>İskenderun, Hatay TÜRKIYE
Keywords:	Needle Fish; systematic; belone species; COI gene; 12s rRNA gene
Abstract:	In this study, we aimed to clarify the taxonomic status of Belonidae species distributed in the Mediterranean Sea and the Black Sea by conducting detailed genetic and morphological markers. A total of 550 needlefish samples were caught between January 2022 and January 2024. The data set used in the study contains a total of 171 sequences for the COI gene and 120 sequences for the 12s rRNA gene from different Belonidae species, including data from GenBank. systematic analysis of needlefish species was investigated by using mtDNA COI and 12s rRNA gene region sequencing and morphological characters in the Turkish Marine Waters. A separate analysis of the two mitochondrial genes supported by morphological characters revealed that each species is grouped within itself. the genetic and morphological analyses showed that Belone belone acus and Belone belone euxini which are considered as the subspecies of Belone belone are not subspecies of the genus Belone and should be considered at the species level, Belone belone. Belone svetovidovi also considerably differs from Belone belone and be considered as a different species. T. acus imperialis, which is thought to be distributed in the Mediterranean Sea, is not a subspecies of Tylosorus acus. Tylosorus imperialis differs from Tylosorus acus and also other Tylosorus species at the species level. These results suggest that the species, which was previously considered as T. acus imperialis from the Mediterranean, should be evaluated at the species level as Tylosurus imperialis due to its high genetic differentiation.
Order of Authors:	Cemal Turan, PhD
	Petya Pavlova Ivanova
	Servet Ahmet DOĞDU, Asisst. Prof. Dr.
	Deniz ERGÜDEN, Prof. Dr.
	Violin Stoyanov Raykov, Assost. Prof. Dr.
	Maria Yankova
	Deniz YAĞLIOĞLU, Prof. Dr.
	Ayşegül ERGENLER
Additional Information:	
Question	Response
Financial Disclosure<br><br>Enter a financial disclosure statement that describes the sources of funding for the work included in this submission. Review the submission guidelines for detailed	Yes

Powered by Editorial Manager® and ProduXion Manager® from Aries Systems Corporation

requirements. View published research articles from *PLOS ONE* for specific examples.<br><br>This statement is required for submission and **will appear in the published article** if the submission is accepted. Please make sure it is accurate.<br><br>**Funded studies**<br>Enter a statement with the following details:<br>• Initials of the authors who received each award<br>• Grant numbers awarded to each author<br>• The full name of each funder<br>• URL of each funder website<br>• Did the sponsors or funders play any role in the study design, data collection and analysis, decision to publish, or preparation of the manuscript?<br><br>Did you receive funding for this work?	
Please add funding details.<br>as follow-up to "**Financial Disclosure**<br><br>Enter a financial disclosure statement that describes the sources of funding for the work included in this submission. Review the submission guidelines for detailed requirements. View published research articles from *PLOS ONE* for specific examples.<br><br>This statement is required for submission and **will appear in the published article** if the submission is accepted. Please make sure it is accurate.	This research was supported by the Scientific & Technological Research Council of Turkey (TUBITAK-121N777) and Bulgarian Academy of Sciences (BAS-IC-TR/1/2022-2023). The authors thank to TUBITAK for their supports.

**Funded studies**<br>Enter a statement with the following details:<br>• Initials of the authors who received each award<br>• Grant numbers awarded to each author<br>• The full name of each funder<br>• URL of each funder website<br>• Did the sponsors or funders play any role in the study design, data collection and analysis, decision to publish, or preparation of the manuscript?<br><br>Did you receive funding for this work?"	
Please select the country of your main research funder (please select carefully as in some cases this is used in fee calculation).<br>as follow-up to "**Financial Disclosure**<br><br>Enter a financial disclosure statement that describes the sources of funding for the work included in this submission. Review the submission guidelines for detailed requirements. View published research articles from *PLOS ONE* for specific examples.<br><br>This statement is required for submission and **will appear in the published article** if the submission is accepted. Please make sure it is accurate.<br><br>**Funded studies**<br>Enter a statement with the following details:<br>• Initials of the authors who received each award<br>• Grant numbers awarded to each author<br>• The full name of each funder<br>• URL of each funder website<br>• Did the sponsors or funders play any role in the study design, data collection and analysis, decision to publish, or preparation of the manuscript?<br><br>Did you receive funding for this work?"	TURKEY - TR
**Competing Interests**	The authors have declared that no competing interests exist.

Use the instructions below to enter a competing interest statement for this submission. On behalf of all authors, disclose any competing interests that could be perceived to bias this work—acknowledging all financial support and any other relevant financial or non-financial competing interests.<br><br>This statement is required for submission and **will appear in the published article** if the submission is accepted. Please make sure it is accurate and that any funding sources listed in your Funding Information later in the submission form are also declared in your Financial Disclosure statement.<br><br>View published research articles from *PLOS ONE* for specific examples.<br><br>**NO authors have competing interests**<br><br>Enter: *The authors have declared that no competing interests exist.*<br><br>**Authors with competing interests**<br><br>Enter competing interest details beginning with this statement:<br><br>*I have read the journal's policy and the authors of this manuscript have the following competing interests: [insert competing interests here]*<br><br>* typeset	
**Ethics Statement**<br><br>Enter an ethics statement for this submission. This statement is required if the study involved:<br><br>• Human participants<br>• Human specimens or tissue<br>• Vertebrate animals or cephalopods<br>• Vertebrate embryos or tissues	N/A

Powered by Editorial Manager® and ProduXion Manager® from Aries Systems Corporation

• Field research<br><br>Write "N/A" if the submission does not require an ethics statement.<br><br>General guidance is provided below. Consult the submission guidelines for detailed instructions. **Make sure that all information entered here is included in the Methods section of the manuscript.**	

Powered by Editorial Manager® and ProduXion Manager® from Aries Systems Corporation

**Format for specific study types**<br><br>**Human Subject Research (involving human participants and/or tissue)**<br>• Give the name of the institutional review board or ethics committee that approved the study<br>• Include the approval number and/or a statement indicating approval of this research<br>• Indicate the form of consent obtained (written/oral) or the reason that consent was not obtained (e.g. the data were analyzed anonymously)<br><br>**Animal Research (involving vertebrate animals, embryos or tissues)**<br>• Provide the name of the Institutional Animal Care and Use Committee (IACUC) or other relevant ethics board that reviewed the study protocol, and indicate whether they approved this research or granted a formal waiver of ethical approval<br>• Include an approval number if one was obtained<br>• If the study involved *non-human primates*, add *additional details* about animal welfare and steps taken to ameliorate suffering<br>• If anesthesia, euthanasia, or any kind of animal sacrifice is part of the study, include briefly which substances and/or methods were applied<br><br>**Field Research**<br><br>Include the following details if this study involves the collection of plant, animal, or other materials from a natural setting:<br>• Field permit number<br>• Name of the institution or relevant body that granted permission	
**Data Availability**<br><br>Authors are required to make all data underlying the findings described fully available, without restriction, and from the time of publication. PLOS allows rare exceptions to address legal and ethical concerns. See the PLOS Data Policy and FAQ for detailed information.	Yes - all data are fully available without restriction

A Data Availability Statement describing where the data can be found is required at submission. Your answers to this question constitute the Data Availability Statement and **will be published in the article**, if accepted.<br><br>**Important:** Stating 'data available on request from the author' is not sufficient. If your data are only available upon request, select 'No' for the first question and explain your exceptional situation in the text box.<br><br>Do the authors confirm that all data underlying the findings described in their manuscript are fully available without restriction?	
**Describe where the data may be found in full sentences. If you are copying our sample text, replace any instances of XXX with the appropriate details.**<br><br>• If the data are **held or will be held in a public repository**, include URLs, accession numbers or DOIs. If this information will only be available after acceptance, indicate this by ticking the box below. For example: *All XXX files are available from the XXX database (accession number(s) XXX, XXX.*).<br>• If the data are all contained **within the manuscript and/or Supporting Information files**, enter the following: *All relevant data are within the manuscript and its Supporting Information files.*<br>• If neither of these applies but you are able to provide **details of access elsewhere**, with or without limitations, please do so. For example:<br><br>*Data cannot be shared publicly because of [XXX]. Data are available from the XXX Institutional Data Access / Ethics Committee (contact via XXX) for researchers who meet the criteria for access to confidential data.*<br><br>*The data underlying the results presented in the study are available from (include the name of the third party*	All files are available from the corrosponding author.

and contact information or URL).<br>• This text is appropriate if the data are owned by a third party and authors do not have permission to share the data.<br><br>* typeset	
Additional data availability information:	Tick here if your circumstances are not covered by the questions above and you need the journal's help to make your data available.

Manuscript
Click here to access/download;Manuscript;MS_Belonidae_Systematic.doc

Systematic Identification of Needle Fish (Belonidae) Species using Molecular Genetic and Morphological Markers in the Mediterranean Sea

Cemal Turan[1*], Petya Pavlova Ivanova[2], Servet Ahmet Doğdu[1,3], Deniz Ergüden[1], Violin Stoyanov Raykov[2], Maria Yankova[2], Deniz Yağlioğlu[4], Ayşegül Ergenler[1]

[1] *Molecular Ecology and Fisheries Genetics Laboratory, Faculty of Marine Sciences and Technology, Iskenderun Technical University, 31200 Iskenderun, Hatay, Türkiye.*

[2] *Marine Biology and Ecology Department, Institute of Oceanology, Bulgarian Academy of Sciences, First May Street 40, P.O. Box 152, Varna, 9000, Bulgaria*

[3] *Iskenderun Technical University, Maritime Technology Vocational School of Higher Education, Underwater Technologies, 31200 Iskenderun, Hatay, Türkiye.*

[4] *Department of Biology, Faculty of Arts and Sciences, Duzce University, 81620 Duzce, Turkey*

*Corresponding author
E-mail: cemal.turan@iste.edu.tr

Abstract

In this study, we aimed to clarify the taxonomic status of Belonidae species distributed in the Mediterranean Sea and the Black Sea by conducting detailed genetic and morphological markers. A total of 550 needlefish samples were caught between January 2022 and January 2024. The data set used in the study contains a total of 171 sequences for the COI gene and 120 sequences for the 12s rRNA gene from different Belonidae species, including data from GenBank. systematic analysis of needlefish species was investigated by using mtDNA COI and 12s rRNA gene region sequencing and morphological characters in the Turkish Marine Waters. A separate analysis of the two mitochondrial genes supported by morphological characters revealed that each species is grouped within itself. the genetic and morphological analyses showed that *Belone belone acus* and *Belone belone euxini* which are considered as the subspecies of *Belone belone* are not subspecies of the genus *Belone* and should be considered at the species level, *Belone belone*. *Belone svetovidovi* also considerably differs from *Belone belone* and be considered as a different species. *T. acus imperialis*, which is thought to be distributed in the Mediterranean Sea, is not a subspecies of *Tylosorus acus. Tylosorus imperialis* differs from *Tylosorus acus* and also other *Tylosorus species* at the species level. These results suggest that the species, which was previously considered as *T. acus imperialis* from the Mediterranean, should be evaluated at the species level as *Tylosurus imperialis* due to its high genetic differentiation.

Keywords: *Needle Fish, systematic, belone species, COI gene, 12s rRNA gene*

Introduction

The family Belonidae, occurring in all tropical and temperate seas, is characterized by their long bodies, long beaks with sharp teeth, and the position of their dorsal and anal fins, and represents 10 genera and 46 valid species worldwide (Fricke et al., 2024). The family are epipelagic, feeding near the surface and has a migratory pattern similar to the mackerel species (Muus and Nielsen, 1999; Froese and Pauly, 2024). Four of the species (*Belone belone*, *B. svetovidovi*, *Tylosurus imperialis and T. choram*) inhabit the Mediterranean Sea (Golani et al., 2006; Froese and Pauly, 2024), and three of which are distributed in the eastern Mediterranean and Black Sea (Karataş et al., 2021; Turan et al., 2023). The garfish *Belone belone* (Linnaeus, 1760) is distributed in the eastern Atlantic Ocean and the Mediterranean Sea (Collette, 2003). Three sub-species have been identified (Collette and Parin, 1986). *B. belone belone* (Linnaeus, 1760) is restricted to the northeastern Atlantic. *B. belone gracilis* Lowe, 1839 is distributed from the south of France in the Mediterranean Sea to the Canary Islands in the Atlantic. *B. belone euxini* Günther, 1866 is distributed in the Black Sea and the Sea of Azov (Yankova et al., 2023; Froese and Pauly, 2024)

The short-beaked garfish *Belone svetovidovi* is a benthic fish species and prefers tropical The distribution area of this species in the eastern Atlantic Ocean extends to Southern Ireland, Spain, Portugal, Israel and Turkey (Dorman, 1987; Meriç and Altun, 1999; Froese and Pauly, 2024). In recent years, it has also been reported to be distributed in the Marmara Sea and the Black Sea (Turan et al., 2023).

The needlefish *Tylosurus acus* (Lacepède, 1803) is an epipelagic species distributed globally in tropical and subtropical waters (Imsiridou et al., 2016), having a considerable number of sub-species (Collette, 2003; Imsiridou et al., 2016). Collette and Parin (1970) identified five sub-species of *Tylosurus acus*; *Tylosurus acus acus* (Lacepède 1803) in the western Atlantic, *T. a. imperialis* (Rafinesque 1810) in the Mediterranean Sea, *T. a. rafale* Collette & Parin 1970 in the Gulf of Guinea, *T. a. melanotus* (Bleeker 1850) throughout the Indo-western Pacific and extending into the eastern Pacific, and *T. a. pacificus* (Steindachner 1876) in the eastern Pacific. The latter subspecies was subsequently raised to species level as *Tylosurus pacificus* by Collette and Banford (2001).

There are many studies on the biological parameters of these species described as species or sub-species level, *Belone belone* (Uçkun et al., 2004; Samsun and Sağlam, 2021; Châari et al., 2022; Yankova et al., 2023; D'Iglio et al., 2024), *Belone belone euxini* (Samsun et al., 2006). Few studies have been conducted on the biology of *Belone svetovidovi* (Bilge et al., 2014) and *Tylosurus acus imperialis* (Chaari et al., 2014; 2016). There have also been several genetic studies on the phylogeny of the Belonidae family. Lovejoy (2000) studied the phylogenetic relationships among different genera of needlefishes, sauries, halfbeaks and flying fishes using mitochondrial, nuclear and morphological characters to better understand the evolutionary changes in the jaw ontogeny of needlefishes. Collete and Banford (2001) compared *Tyleserus* genera with ATPase 8,6 and cyt b gene regions in their study of Pacific stingfish populations and found that the subspecies *Tylosurus acus pacificus* differed morphologically and genetically from *Tylosurus acus,* and concluded that *T. pacificus* should be registered as a species. Imsiridou et al. (2016) carried out the genetic and phenotypic identification of *Tylosurus acus imperialis* specimens from the North Aegean Sea using 16s rRNA gene region and reported that *T. a. imperialis* is genetically and morphologically different from *T. choram* and *T. crocodilus*. Yankova et al. (2023) analysed the genetic and morphometric characteristics of the *Belone belone* population on the Nesebar coast of Bulgaria and reported that *B. belone* experienced a population decline due to overfishing on the Bulgarian coast.

Phylogenetic analyses of fish species are fundamental to taxonomy as they facilitate the understanding and classification of evolutionary relationships among fish species. Phylogenetic analyses can include data from three different domains: morphological (phenotypic characteristics), molecular (genetic structures) and behavioural (Nelson et al., 2016). Through these analyses, fish species can be grouped with similar characteristics, and phylogenetic trees can be created to determine the evolutionary relationships among different species (Near et al., 2013). Therefore, the phylogenetic analyses enable us to make accurate classifications in fish taxonomy and better understand the relationships among species.

In this study, it was aimed to clarify the taxonomic status of Belonidae species distributed in the Mediterranean and Black Sea by conducting detailed genetic and morphological markers.

Material and Methods

Sampling

A total of 550 needlefish samples were caught between January 2022 and January 2024. Of these putative sampling, 50 samples were *Belone belone* (25 Nesebar and 25 Akçakoca), 350 samples were *B. svetovidovi* (50 Muğla, 50 İzmir, 50 Çanakkale, 50 Yalova, 50 Düzce, 50 Sinop and 50 Rize coasts) and 150 samples were *T.a. imperialis* species (İskenderun, Mersin and Antalya coasts) (Fig 1). All samples were examined morphologically, and muscle and/or fin tissues were taken for genetic analyses. Identification of the specimens was done following Collete & Parin (1986). This study does not require Local Ethics Committee Approval as experimental animals were not used in this study.

Fig 1. Sampling location. Circle: *T. imperialis*, Squere: *Belone svetovidovi*, Trigle: *Belone belone*.

Genetic Analysis

The specimens were delivered to the laboratory and stored in a deep freeze at -30 °C until DNA extraction. Total genomic DNA was extracted from muscle or fin tissues using a modified phenol-chloroform isoamyl alcohol method (Sambrook et al. 1989). The mtDNA COI and 12s rRNA gene regions were amplified through PCR with universal primers (Kocher et al., 1989; Ward et al., 2005).

Table 1. Universal Primers of COI and 12s rRNA gene regions.

**COI**	COI_F:	5’-TCA ACC AAC CAC AAA GAC ATT GGC AC-’3’
	COI_R:	5’-ACT TCA GGG TGA CCG AAG AAT CAG AA-’3’
**12s rRNA**	12s_F:	5’- CAA ACT GGG ATT AGA TAC CCC ACT AT -3’
	12s_R:	5’- GAG GGT GAC GGG CGG TGT GT -3’

The PCRs were conducted in a 50 µl total volume with 0.4 uM of each primer, 0.2 mM of dNTP and 1 U of Taq DNA polymerase in a PCR buffer that included 20 mM of Tris–HCl (pH 8.0), 1.5 mM of MgCl2, 15 mM of KCl and 1.5 µl template DNA. The denaturation step was at 94°C for 30 s, 60°C for 30 s, and 75°C for 45 s for 35 cycles followed by a final extension for 7 min at 75 °C. The PCR products were visualized using electrophoresis on 1.5 % agarose gel. DNA sequencing was performed by chain termination method by Sanger et al. (1977) with Bigdye Cycle Sequencing Kit V3.1 and ABI 3130 XL genetic analyzer. The DNA sequence analyses were performed on 10 sample specimens of various sizes from each population. The initial alignments of partial mtDNA sequences were performed with the Clustal W (Thompson et al., 1994), and the final alignment was completed manually with BioEdit (Hall, 1999). Haplotypes diversity, genetic diversity (Nei, 1987), and the mean number of pairwise differences (Tajima, 1983) were calculated using the program of Arlequin (Schneider et al. 2000). Haplotypes were obtained with the DnaSP 6 program (Rozas, 2017). The minimum spanning tree (MST) (Excoffier & Peter, 1996) of haplotypes was revealed with the Popart (Leigh et al., 2015).

For COI gene regions, a total of 120 specimens, 20 *B. belone* specimens from two populations, 70 *B. svetovidovi* specimens from seven populations, and 30 *T. imperialis* specimens from three populations, were sequenced for the mtDNA COI gene region. For 12s rRNA gene regions, a total of 110 specimens, 10 *B. belone* samples from one population, 70 *B. svetovidovi* specimens from seven populations, and 30 *T. imperialis* specimens from three populations, were sequenced for the mtDNA 12s rRNA gene region. The data set used in the study contains a total of 171 sequences for the COI gene and 120 sequences for the 12s rRNA gene from different Belonidae species, including data from GenBank (see Appendix 1). Furthermore, the species of *Ablennes hians* (Valenciennes, 1846) was used as an outgroup as it belongs to the Belonidae family. Phylogenetic relationships were estimated with the MEGAX software (Kumar et al., 2018), using the maximum likelihood (ML) and maximum parsimony (MP) phylogenetic tree analyses.

Morphological analysis

Morphometric measurements were carried out on the photographs of the fish samples using the BioMorph software (Kutlu and Turan 2018). The morphometric characters analysed are presented in Fig 2.

Fig 2. Morphometric measurements of needlefish. TL: total length, FL: fork length, SL: standard length, PreD: Predorsal distance, PostD: Postdorsal distance, PreA: preanal distance, PostA: Postanal distance, PreV: preventral distance, HL: head length, 12-13: eye diameter, H: maximum body height, h: minimum body height, 3-4: dorsal fin base length, 8-9: anal fin base length, 14-15: pectoral fin length, 10-18: ventral fin length (Figure: Collette, 2016).

A principal component analysis (PCA) and discriminant function analysis (DFA) were performed for the classification of species based on morphometric characters. Most of the variability in morphological characters is due to size differences (Junquera and Perez-Gandaras, 1993). Therefore, shape analysis should be free from the effects of size variation to avoid misinterpretation of the results (Strauss, 1985). Therefore, the first principal component of PCA was removed from the analyses since it represents a multivariate generalization of simple allometry (Jolicoeur, 1963; Klingenberg, 1996), subsequent components were used for multivariate morphometric analysis. A Hierarchical cluster analysis using Euclidean distance was performed to see morphological relationships among species. All calculations were performed in SPSS, SYSTAT and R Studio programs.

Results

COI gene

Polymorphisms within the mtDNA COI gene region were obtained as 113 variable and 493 conservative nucleotides of which 106 were parsimony informative over 606 bp sequences. The best model (HKY+G) was provided by the MEGAX software (Hall, 2013). The parameters of this model were: nucleotide frequencies A: 26.2%, T: 33.0%, C: 25.10%, G: 16.10%, +G: 0.19. The estimated Transition/Transformation bias (R) was found to be 2.07.

Twenty-eight haplotypes were found out of 120 sequences, and there were no common haplotypes between the species (Table 2). Haplotype diversity was found to be 0.8594. The minimum spanning tree (MST) analysis revealed a high amount of mutation between the species (Fig 3).

Table 2. Haplotype number and its distribution between the species for COI gene region.

**Haplotypes**	***Belone belone***	***Belone svetovidovi***	***Tylosurus imperialis***	**Total**
**Hap_1**	**10**	-	-	**10**
**Hap_2**	**10**	-	-	**10**
**Hap_3**	-	**41**	-	**41**
**Hap_4**	-	**5**	-	**5**
**Hap_5**	-	**3**	-	**3**
**Hap_6**	-	**1**	-	**1**
**Hap_7**	-	**1**	-	**1**
**Hap_8**	-	**1**	-	**1**
**Hap_9**	-	**1**	-	**1**
**Hap_10**	-	**1**	-	**1**
**Hap_11**	-	**1**	-	**1**
**Hap_12**	-	**1**	-	**1**
**Hap_13**	-	**1**	-	**1**
**Hap_14**	-	**1**	-	**1**
**Hap_15**	-	**1**	-	**1**
**Hap_16**	-	**7**	-	**7**
**Hap_17**	-	**3**	-	**3**
**Hap_18**	-	**1**	-	**1**
**Hap_19**	-	-	**4**	**4**
**Hap_20**	-	-	**1**	**1**
**Hap_21**	-	-	**9**	**9**
**Hap_22**	-	-	**5**	**5**
**Hap_23**	-	-	**2**	**2**
**Hap_24**	-	-	**2**	**2**
**Hap_25**	-	-	**1**	**1**
**Hap_26**	-	-	**2**	**2**
**Hap_27**	-	-	**2**	**2**
**Hap_28**	-	-	**2**	**2**
**Total**	**20**	**70**	**30**	**120**

Fig 3. Minimum spanning tree for the COI gene region, showing the evolutionary mutation relationships between haplotypes.

The mean differentiation (F_{ST}) between individuals of a species was found to be the lowest in *B. belone* and the highest in *B. svetovidovi* (Table 3). In pairwise comparison of species, the F_{ST} values ranged from 0.0345 to 0.1699 (Table 3). The lowest genetic distance was observed between *B. belone* and *B. svetovidovi* (0.0345) and the highest genetic distance was observed between *B. svetovidovi* and *T. imperialis* (0.1699) species.

Table 3. The lower part of the table is given for the COI gene region. In bold are the within-species genetic divergence matrix and pairwise genetic distances between species that are statistically significant for the COI gene region. The upper part of the table is for the 12s rRNA gene region. Intraspecific genetic divergence matrix in bold and italic letters and pairwise genetic distances between species that are statistically significant for the 12s rRNA gene region are given in italics (*; P<0.001).**

Species	*B. belone*	*B. svetovidovi*	*T. İmperialis*
B. belone	**0.0017/*0.0001***	*0.0050****	*0.0832****
B. svetovidovi	0.0345***	**0.0036/*0.0016***	*0.0871****
T. İmperialis	0.1679***	0.1699***	**0.0030/*0.0023***

The Maximum Likelihood (ML) and Maximum-Parsimony (MP) phylogenetic tree analyses revealed a similar tree topology (Fig 4 and Fig 5.). The two species of the genus Belone, *B. belone* and *B. svetovidovi*, were separated from the same branch, while the other species of the genus Tylosurus, *T. imperialis*, was branched as a separate branch. According to ML and MP trees, *B. belone*, *B. svetovidovi* and *T. imperialis* were clustered in different sets, and *Tylosorus imperialis* differs from other *Tylosorus* species at the species level by forming a separate branch with the *Tylosorus acus imperialis* reference sequences. These results prove that the species previously referred to as *T.a. imperialis* from the Mediterranean Sea *is Tylosorus imperialis.*

Fig 4. Maximum Likelihood tree (ML) of needlefish species with reference samples for COI gene region.

Fig 5. Maximum Parsimony tree (MP) of needlefish species with reference samples for COI gene region.

12s rRNA gene

After alignment, there were observed 46 variable and 379 conservative nucleotides of which 40 were parsimony informative over 425 bp sequences obtained from mtDNA 12s rRNA gene region. The best model (K2) was provided by the MEGAX software (Hall, 2013). The parameters of this model were: nucleotide frequencies A: 25 %, T: 25%, C: 25% and G: 25%. The estimated Transition/Transformation bias (R) was found to be 1.51

Twenty-three haplotypes were found from a total of 110 sequences. and there were no common haplotypes between the species (Table 4). Haplotype diversity was found to be 0.7843. The minimum spanning tree (MST) which shows the relationship between haplotypes is given in Fig 6. According to the MST analysis, a high amount of mutation was observed between the species.

Table 4. Haplotype number and its distribution between the species for 12s rRNA gene region.

Haplotypes	*Belone belone*	*Belone svetovidovi*	*Tylosurus imperialis*	Total
**Hap_1**	10	-	-	**10**
**Hap_2**	-	42	-	**42**
**Hap_3**	-	2	-	**2**
**Hap_4**	-	1	-	**1**
**Hap_5**	-	21	-	**21**
**Hap_6**	-	1	-	**1**
**Hap_7**	-	1	-	**1**
**Hap_8**	-	2	-	**2**
**Hap_9**	-	-	2	**2**
**Hap_10**	-	-	19	**19**
**Hap_11**	-	-	1	**1**
**Hap_12**	-	-	3	**3**
**Hap_13**	-	-	1	**1**
**Hap_14**	-	-	1	**1**
**Hap_15**	-	-	1	**1**
**Hap_16**	-	-	1	**1**
**Hap_17**	-	-	1	**1**
**Total**	**10**	**70**	**30**	**110**

Fig 6. Minimum spanning tree for the 12s rRNA gene region, showing the evolutionary mutation relationships between haplotypes.

The mean pairwise differentiation (F_{ST}) within species was found to be the lowest in the B. belone species and the highest in the T. imperialis species (Table 3). In pairwise comparison of species, the F_{ST} values ranged from 0.0050 to 0.0871 (Table 3). The pairwise F_{ST} values showed that some species were significantly distinct from each other ($P<0.05$).

The Maximum Likelihood (ML) and maximum parsimony (MP) phylogenetic tree analyses were given in Fig 7 and Fig 8. NJ and ML trees indicated similar tree topologies. The two species of the genus Belone, *B. belone* and *B. svetovidovi*, were separated from the same branch, while the other species of the genus Tylosurus, *T. imperialis*, was branched as a separate branch. According to NJ and MP trees, *B. belone*, *B. svetovidovi* and *T. imperialis* were clustered in different sets. Also, *the species Tylosorus imperialis* differs from *Tylosorus acus* and other *Tylosorus* species at the species level by forming a separate branch with *Tylosorus imperialis* reference sequences. These results prove that the species previously recorded as *T.a. imperialis* from the Mediterranean Sea *is Tylosorus imperialis.*

Fig 7. Maximum Likelihood tree (ML) of needlefish species with reference samples for 12s rRNA gene region.

Fig 8. Maximum Parsimony tree (MP) of needlefish species with reference samples for 12s rRNA gene region.

Morphology

Discriminative morphometric and meristic characters of sampled species are given in Table 5 and Table 6. *Belone belone* is morphometrically similar to *B. svetovidovi* but has a heavier, less compressed body and wider inter-orbital width. The lower jaw is a little longer than the upper jaw. Teeth are usually present on the vomer in specimens larger than 28 cm in Standard length (SL). Dorsal fin rays 16-20; Anal fin rays 19-23; Pectoral fin rays 11-14. Gill rakers 27-40 on the first arch, Vertebrae 75-84.

Fig 9. *Belone belone* individual (a- *Belone belone*'s general appearance, b- snout and head structure, c- dorsal, anus and caudal fin areas).

Table 5. Relative relationships of measured body proportions of needlefish collected from Turkish Marine Waters and the Bulgarian coast. N: number of fish studied, MV: mean value, SD: Standard deviation, SE: Standard error of mean value; CV: coefficient of variation.

Species	Relation	Sex	N	Range	MV±SE	MV±SD	CV (%)
Belone belone	**FL/TL**	F+M	50	0.94-0.98	0.957±0.001	0.957±0.007	0.73
	**SL/TL**	F+M	50	0.89-0.96	0.920±0.001	0.920±0.012	1.30
	**HL/TL**	F+M	50	0.12-015	0.134±0.001	0.134±0.008	5.97
	**O/TL**	F+M	50	0.02-0.03	0.026±0.000	0.026±0.004	15.38
	**H/HL**	F+M	50	0.05-0.07	0.059±0.000	0.059±0.002	3.38
	**h/TL**	F+M	50	0.01-0.04	0.022±0.000	0.022±0.003	13.63
	**PODL/TL**	F+M	50	0.81-0.87	0.846±0.001	0.846±0.012	1.42
	**PDL/TL**	F+M	50	0.65-0.71	0.680±0.002	0.680±0.014	2.05
	**POAL/TL**	F+M	50	0.81-0.88	0.847±0.002	0.847±0.015	1.77
	**PAL/TL**	F+M	50	0.63-0.70	0.659±0.002	0.659±0.014	2.12
	**PVL/TL**	F+M	50	0.49-0.55	0.518±0.002	0.518±0.015	2.89
	**PPL/TL**	F+M	50	0.11-0.18	0.155±0.001	0.155±0.012	7.74
**Species**	**Relation**	**Sex**	**N**	**Range**	**MV±SE**	**MV±SD**	**CV (%)**
Belone svetovidovi	**FL/TL**	F+M	350	0.94-0.99	0.962±0.000	0.962±0.006	0.62
	**SL/TL**	F+M	350	0.90-0.97	0.924±0.000	0.924±0.008	0.86
	**HL/TL**	F+M	350	0.09-0.17	0.134±0.000	0.134±0.012	8.95
	**O/TL**	F+M	350	0.02-0.04	0.027±0.000	0.027±0.004	14.81
	**H/TL**	F+M	350	0.05-0.08	0.062±0.000	0.062±0.004	6.45
	**h/LH**	F+M	350	0.01-0.03	0.024±0.000	0.024±0.003	12.5
	**POD/TL**	F+M	350	0.81-0.91	0.850±0.000	0.850±0.011	1.29
	**PDL/TL**	F+M	350	0.65-0.76	0.689±0.000	0.689±0.012	1.74
	**POAL/TL**	F+M	350	0.80-0.91	0.845±0.000	0.845±0.012	1.42
	**PAL/TL**	F+M	350	0.63-0.74	0.667±0.000	0.667±0.012	1.80
	**PVL/TL**	F+M	350	0.41-0.57	0.526±0.001	0.526±0.020	3.80
	**PPL/TL**	F+M	350	0.12-0.19	0.149±0.000	0.149±0.013	8.72
**Species**	**Relation**	**Sex**	**N**	**Range**	**MV±SE**	**MV±SD**	**CV (%)**
Tylosuruss imperialis	**FL/TL**	F+M	150	0.93-0.98	0.951±0.000	0.951±0.007	0.73
	**SL/TL**	F+M	150	0.90-0.96	0.926±0.000	0.926±0.008	0.86
	**HL/TL**	F+M	150	0.04-0.07	0.059±0.000	0.059±0.004	6.78
	**O/TL**	F+M	150	0.02-0.04	0.026±0.000	0.026±0.003	11.53
	**H/TL**	F+M	150	0.04-0.07	0.059±0.000	0.059±0.004	6.78
	**h/LH**	F+M	150	0.01-0.03	0.022±0.000	0.022±0.002	9.09
	**POD/TL**	F+M	150	0.85-0.91	0.877±0.000	0.877±0.010	1.14
	**PDL/TL**	F+M	150	0.65-0.75	0.680±0.000	0.680±0.010	1.47

	**POAL/TL**	F+M	150	0.84-0.91	0.872±0.000	0.872±0.009	1.03
	**PAL/TL**	F+M	150	0.63-0.74	0.672±0.000	0.672±0.011	1.63
	**PVL/TL**	F+M	150	0.45-0.55	0.489±±0.001	0.489±±0.012	2.45
	**PPL/TL**	F+M	150	0.12-0.17	0.138±±0.000	0.138±±0.010	7.24

Table 6. Meristic characteristic of needlefish. Dorsal fin ray number (DIS), Anal ray number (AIS), Pectoral fin ray number (PIS), Number of teeth, Number of gill spines, and Number of vertebrae were counted. In addition, data on the gonad status of each specimen were taken. N: number of fish studied, MV: mean value, SD: standard deviation, SE: standard error of mean value, CV: coefficient of variation.

**Species**	**Meristic characters**	**Sex**	**N**	**Range**	**MV±SE**	**MV±SD**	**CV (%)**
Belone belone	**Dorsal fin ray number (DIS)**	F+M	50	16-19	17.125±0.44	17.125±1.24	7.24
	**Anal ray number (AIS)**	F+M	50	21-23	21.750±0.25	21.750±0.71	3.26
	**Pectoral fin ray number (PIS)**	F+M	50	12-13	12.750±0.16	12.750±0.46	3.60
	**Number of upper teeth (UT)**	F+M	50	8-10	8.875±0.29	8.875±0.83	9.35
	**Number of bottom teeth (UT)**	F+M	50	7-9	7.875±0.29	7.875±0.83	10.53
	**Number of gill spines (GS)**	F+M	50	27-39	32.750±1.54	32.750±4.36	13.31
	**Number of vertebrae (V)**	F+M	50	76-81	78.125±0.64	78.125±1.80	2.30
**Species**	**Meristic characters**	**Sex**	**N**	**Range**	**MV±SE**	**MV±SD**	**CV (%)**
Belone svetovidovi	**Dorsal fin ray number (DIS)**	F+M	350	15-20	17.710±0.13	17.710±1.33	7.50
	**Anal ray number (AIS)**	F+M	350	19-23	22.240±0.09	22.240±0.95	4.27
	**Pectoral fin ray number (PIS)**	F+M	350	12-13	12.790±0.04	12.790±0.43	3.36
	**Number of upper teeth (UT)**	F+M	350	11-18	14.140±0.18	14.140±1.89	3.59
	**Number of bottom teeth (UT)**	F+M	350	12-19	15.620±0.16	15.620±1.61	2.62
	**Number of gill spines (GS)**	F+M	350	38-46	41.270±0.20	41.270±2.00	4.84
	**Number of vertebrae (V)**	F+M	350	69-86	77.390±0.47	77.390±4.75	6.13
**Species**	**Meristic characters**	**Sex**	**N**	**Range**	**MV±SE**	**MV±SD**	**CV (%)**
Tylosurus imperialis	**Dorsal fin ray number (DIS)**	F+M	150	19-26	23.471±0.10	23.471±1.21	5.15
	**Anal ray number (AIS)**	F+M	150	18-24	21.300±0.12	21.300±1.33	6.24
	**Pectoral fin ray number (PIS)**	F+M	150	12-16	13.203±0.69	13.203±0.76	5.75
	**Number of upper teeth (UT)**	F+M	150	18-48	30.951±0.60	30.951±6.70	21.64
	**Number of bottom**	F+M	150	15-45	29.081±0.63	29.081±7.00	24.07

	**teeth (UT)**						
	**Number of gill spines (GS)**	F+M	150	0	0	0	0
	**Number of vertebrae (V)**	F+M	150	86-96	91.008±0.19	91.008±2.17	2.38

[illegible]. *sveovidovi*, although very resemble to *B. belone* and previously confused with *Belone belone.* Both jaws are very elongated, Upper teeth are smaller and closer together. At first glance, it differs from *B. belone* in the size and density of its beak teeth. Dorsal fin rays 16-19; Anal fin rays 20-23; Pectoral fin rays 11-13. Gill rakers 38-52 on the first arch, Vertebrae 70-86.

B. sveovidovi can be distinguished from its Mediterranean garfish *B. belone* by the following characteristics: Its smaller, more delicate and features 13-21 teeth within a section of the middle of the upper jaw equaling the diameter of its eye and teeth present on vomer and total gill rakers on first gill arch 38-52 (Akşıray, 1987; Collette & Parin 1990; Dalyan & Eryılmaz, 2005).

Fig 10. *Belone svetovidovi* individual (a- *Belone svetovidovi* 's general appearance, b- snout and head structure, c- dorsal, anus and caudal fin areas).

The bones of *T. imperialis* are not green in color as those of the Belone genus. Both jaws are very elongated, anterior lobes of the dorsal and anal relatively low (respectively 10.5-13.3 and 9.7-11.7 times in body length), and pectoral and pelvic relatively short (respectively 8.0-12.4 and 10.0-14.1 times in body length). There are no teeth on the vomer. There is a narrow and small black lateral keel on each side of the caudal peduncle. The caudal fin is forked, with the lower lobe much longer than the upper lobe. Dorsal fin rays 20-27; Anal fin rays 18-24; Pectoral fin rays 12-14. Gill rakers are absent on the arch, Vertebrae 93-96.

Fig 11. *Tylosurus imperialis* individual (a- *Tylosurus imperialis*'s general appearance, b- snout and head structure, c- dorsal, anus and caudal fin areas).

Multivariate analysis

A total of 16 morphometric measurements (were examined. There were significant differences (P<0.001) for all traits when analysed over all the 3 species. In the PCA, the first principal component represented 38% of between group variability was removed from the analysis. The second and third principal components produced 14% and 12% of between group variability, respectively. Examination of the contribution of each morphometric character to the second and third principal components revealed that the pectoral fin length, predorsal distance, postdorsal distance, and eye diameter, highly contributed to the morphometric differentiation of species (Fig 12).

Fig 12. The contribution of each morphometric character to the second and third principal components

Hierarchical canonical analysis shows that *T. imperialis* is quite distinct, with a closer morphological relationship between *B. belone* and *B. svetovidovi* (Fig 13)

Fig 13. Hierarchical canonical analysis of needle fish species.

Discussion

In the present study, systematic analysis of needlefish species was investigated by using mtDNA COI and 12s rRNA gene region sequencing and morphological characters in the Turkish Marine Waters. A separate analysis of the two mitochondrial genes supported by morphological characters revealed that each species is grouped within itself.

The maximum likelihood topology revealed in our study was almost similar to two gene regions (Fig 4 and Fig 7). *T. imperialis* individuals formed a separate clade with a high bootstrap value of 99%, while *T. acus* and *T. a melanatus* subspecies clustered together to form a separate group. *B. belone* and *B. svetovidovi* species were also clustered in separate clades. The present findings for the systematic classification of three species *B. belone*, *B. svetovidovi* and *T. imperialis* found in Turkish marine waters were incongruent with the study by Banford et al. (2004) studied phylogenetic relationships of the Belonid genera. Imsiridou et al. (2016) studied genetic and phenotypic characters using 16s rRNA gene region to identify *T. a. imperialis* in Thermaikos Gulf, North Aegean Sea. In the results obtained, *T. acus* and *T. a. imperialis* were grouped in the same clade. The results of our study show that B. belone and *B. svetovidovi* also form separate clades and are separated from each other with high bootstrap values. The maximum parsimony topology is similar to the Maximum Likelihood tree in both genes (Fig 5 and Fig 8). *T. imperialis*, *B. belone* and *B. svetovidovi* are separated from the other species by distinct clades. Moreover, ML and MP trees constructed with the 12s rRNA gene region show that subspecies of *Tylosorus acus* such as *T. a. melenatus, T.a. rafale*, *T. a. acus* and *T. a. pacificus* cluster together, indicating genetic integrity.

When we look at the minimum spanning tree for both gene regions, it is seen that the species are separated from each other by many mutations (Fig 3 and Fig 6). Especially when we look at the MST of the COI gene region, the species are separated from each other by mutating many times. Each species has formed a separate group and is separated from each other. In the MST of the 12s rRNA gene region, it is seen that fewer mutations occur compared to the COI gene region (Fig 6).

In this study, it was determined that these species differ in terms of the number of gill rakers (27-39 in *B. belone* and 38-46 in *B. svetovidovi*) and vertebrae numbers (76-81 in *B. belone* and 69-86 in *B. belone* and) while there was no meristic difference in terms of the number of dorsal fin rays (16-19 in *B. belone* and 15-20 in *B. svetovidovi*), anal fin rays (21-23 in *B. belone* and 19-23 in *B. svetovidovi*) pectoral fin rays (12-13 in *B. belone* and 12-13 in *B. svetovidovi*). Dorman (1987) examined the morphological characteristics of *Belone belone* and *Belone svetovidovi* species in Northern Europe between 1982 and 1985 and determined the number of gill rakers in the first arch as 30-45 for *B. belone* and 41-53 for *B. belone*. With the number of vertebrae 76-85 for *B. belone* and 76-81 for *B. svetovidovi*. Meriç and Altun (1999) reported most distinct differences separating the two species (B. belone and B. svetovidovi) are the gill-rakers on the first branchial arch: B. svetovidovi have 39-53 gill-rakers, whereas *B.* belone has only 25-37. Moreover, the gill-rakers of *B.* svetovidovi are longer (Table 6).

Collette and Parin (1986) stated that *B. belone* has 3 subspecies. Each of them is distributed in different regions: *B. belone belone* (Linnaeus, 1761) from the north of France to the northeast of the Atlantic, *B. belone euxini* (Günther, 1866) from the Black Sea and the Sea of Azov; The other subspecies, *B. belone gracilis* (Lowe, 1839), is found in the northwestern Atlantic and the Mediterranean waters. These three subspecies are distinguished by their meristic characters such as the number of vertebrae, and the number of fin rays in the dorsal fin (Collette and Parin, 1986). Another species, *Belone svetovidovi* (Collette and Parin, 1970), is distributed in the northeastern Atlantic (south of Ireland, Spain, and Portugal) and the Mediterranean (Collette and Parin, 1986).

Although *Belone belone* and *Belone svetovidovi* species are morphologically similar to each other, *t*he most obvious differences between *B. belone* and *B. svetovidovi* are the size and density of the beak teeth and the number of gills rakers (*Collette and Parin, 1986). B. belone* has body compressed and vomerine teeth usually present in larger specimens but frequently absent in specimens less than 20 cm BL (28 cm SL). Jaw teeth are comparatively large and widely spaced (6-15 in a distance equal to an orbit's length on the middle of the upper jaw). Dorsal fin rays 16-20; Anal fin rays 19-23; Pectoral fin rays 11-14. Gill rakers 27-40 on first arch and Vertebrae number; 75-84. However, *B. svetovidovi* has a body with a more compressed and narrower inter-orbital width. *B. svetovidovi* is characterized by its smaller, more delicate and features 13-21 teeth within a section of the middle of the upper jaw equaling the diameter of its *eye* present on the vomer. Dorsal fin rays 16-19; Anal fin rays 20-23; Pectoral fin rays 11-13. Gill rakers on first gill arch 38-52. Vertebrae number; 70-86 (Collett and Parin, 1970).

Consequently, in the present study, we found that the number of gill-rakers among the meristic characters is the best for different *B. belone* from *B. svetovidovi* (Table 7).

Table 7. The meristic features of *B. belone* and *B. svetovidovi*

Species	References	DFR	AFR	PFR	Teeth (Upper Jaw)	Gill Rakers	Vertebrae
B. belone	Dieuzeide (1954)	16-19	20-22	12-15	-	-	-
B. belone	Dorman (1987)	15-20	17-23	12-14	-	30-45	76-85
B. belone	Meriç & Altun (1999)	16-19	20-22	-	5-10	25-37	76-80
B. belone	Golani et al. (2006)	16-19	19-23	11-14	<13	-	-
B. belone	This study	16-19	21-23	12-13	-	27-39	76-81

B. svetovidovi	Dorman (1987)	16-18	20-23	11-13	-	42-53	76-81
B. svetovidovi	Meriç & Altun (1999)	15-19	19-23	-	11-21	39-53	76-81
B. svetovidovi	Dalyan & Eryılmaz (2006)	17	22	13	14	41	-
B. svetovidovi	Golani et al. (2006)	16-19	20-23	11-13	-	-	-
B. svetovidovi	This study	15-20	19-23	12-13	-	38-46	69-86

Tylosurus imperialis has mostly been accepted as a subspecies of *T. acus imperialis* in the literature (Collette and Parin, 1990), recently, it has been considered as a provisionally accepted name until more works are conducted on *T. acus* subspecies (Froese and Pauly, 2024).

Although these four *Tylosurus* species are morphologically similar to each other, they differ from each other in terms of the number of vertebrae. Total number of vertebrae 93-96 in *T. imperialis*, 75-80 in *T. crocodilus* and 77 in *T. choram*) (Table 8).

Table 8. The meristic features of Tylosurus *species.*

Species	References	DFR	AFR	PFR	Teeth (Upper Jaw)	Gill Rakers	Vertebrae
T. acus	Paugy, 2003	20-27	20-24	-	-	0	90-95
T. choram	Collette & Parin (1986)	19-24	19-22	-	-	0	-
T. choram	Golani et al. (2006)	20-24	19-22	12-14	-	0	-
T. charam	Golani & Levi (2005)	20	20	13	-	0	77
T. crocodilus	Collette (1984)	21-24	19-22	-	-	0	-
T. crocodilus	Collette (1999)	20-24	19-22	12-14	-	0	75-80
T. crocodilus	Collette (2016)	21-23	18-22	13-15	-	0	79-84
T. a. imperialis	Collette & Parin (1986)	23-26	22-23	-	-	0	-
T. a.	Golani et al. (2006)	20-27	18-24	12-14	-	0	-

imperialis							
T. imperialis	This study	19-26	18-24	12-16	18-48	0	86-96

In conclusion, the genetic and morphological analyses showed that *Belone belone acus* and *Belone belone euxini* which are considered as the subspecies of *Belone belone* are not subspecies of the genus *Belone* and should be considered at the species level, *Belone belone*. *Belone svetovidovi* also considerably differs from *Belone belone* and be considered as a different species. *T. acus imperialis*, which is thought to be distributed in the Mediterranean Sea, is not a subspecies of *Tylosorus acus*. *Tylosorus imperialis* differs from *Tylosorus acus* and also other *Tylosorus species* at the species level. These results suggest that the species, which was previously considered as *T. acus imperialis* from the Mediterranean, should be evaluated at the species level as *Tylosurus imperialis* due to its high genetic differentiation.

Acknowledgements

This research was supported by the Scientific & Technological Research Council of Turkey (TUBITAK-121N777) and Bulgarian Academy of Sciences (BAS-IC-TR/1/2022-2023). The authors thank to TUBITAK for their supports.

References

Altin, A., Ayyildiz, H., Kale, S., & Alver, C. (2015). Length-weight relationships of forty-nine fish species from shallow waters of Gökçeada Island, northern Aegean Sea Turkey. Turkish Journal of Zoology, 39(5), 971-975.

Banford, H. M., Bermingham, E., & Collette, B. B. (2004). Molecular phylogenetics and biogeography of transisthmian and amphi-Atlantic needlefishes (Belonidae: Strongylura and Tylosurus): perspectives on New World marine speciation. Molecular Phylogenetics and Evolution, 31(3), 833-851.

Bilge, G., Yapıcı, S., Filiz, H., & Cerim, H. (2014). Weight–length relations for 103 fish species from the southern Aegean Sea, Turkey. Acta ichthyologica et piscatoria, 44(3), 263-269.

Bilgin, S., Taşçi, B., & Bal, H. (2014). Population dynamics of the garfish, Belone euxini (Belonidae: Belone) from the south-east Black Sea. Journal of the Marine Biological Association of the United Kingdom, 94(8), 1687-1700.

Ceyhan, T., Samsun, O., & Akyol, O. (2019). Age, growth and mortality of garfish, Belone euxini Günther, 1866 in the Central Black Sea, Turkey.

Châari, M., Boudaya, L., & Neifar, L. (2016). Preliminary observations on the feeding habits of the Mediterranean needlefish Tylosurus acus imperialis (Teleostei: Belonidae). J Coast Life Med, 4(6), 426-429.

Châari, M., Boudaya, L., Gancitano, S., Gancitano, V., & Neifar, L. (2022). Age, Growth and Reproductive Biology of the Garfish, Belone belone (Linnaeus, 1760)(Teleostei:

Belonidae) in the Central Mediterranean Sea. Turkish Journal of Fisheries and Aquatic Sciences, 22(8).

Chaari, M., Boudaya, L., GanCitano, S., GanCitano, V., Fiorentino, F., & Neifar, L. (2014). First information on biology of the needlefish Tylosurus acus imperialis (Belonidae) off the Tunisian coast (Central Mediterranean). Cybium, 38(4), 273-278.

Çoker, T., Akyol, O., & Bilge, G. (2013). Determination of batch fecundity of garfish, Belone belone, in the northern Aegean Sea. Journal of Black Sea/Mediterranean Environment, 19(3), 385-392.

Çolakoğlu, S. (2021). Length-weight relationships of fishes caught by stationary uncovered pound nets in the coastal waters of Saros Bay, North Aegean Sea (Turkey). Ege Journal of Fisheries and Aquatic Sciences, 38(1), 95-99.

Collette B.B. (1984). Belonidae. In: FAO species identification sheets for fishery purposes. Western Indian Ocean; (Fishing Area 51), Vols 1-6 (W. Fischer & G. Bianchi eds), pag. var. Prepared and printed with the support of the Danish International Development Agency (DANIDA). Food and Agricultural Organization of the United Nations: Rome.

Collette, B.B. (1999). Belonidae. Needlefishes. p. 2151-2161. In K.E. Carpenter and V.H. Niem (eds.). FAO species identification guide for fishery purposes. The living marine resources of the Western Central Pacific. Volume 4. Bony fishes part 2 (Mugilidae to Carangidae). FAO, Rome.

Collette, B. B. 2003. Family Belonidae Bonaparte 1832: needlefishes. California Academy of Sciences Annotated Checklists of Fishes 16:1–22.

Collette, B. B. (2016) Belonidae. In: Carpenter KE, De Angelis N (Eds) The living marine resources of the Eastern Central Atlantic. Volume 3: Bony fishes part 1 (Elopiformes to Scorpaeniformes). FAO Species Identification Guide for Fishery Purposes. FAO, Rome, 914 pp.

Collette, B. B. & Parin, N. V. (1970). Needlefishes (Belonidae) of the eastern Atlantic Ocean. Atlantide Report. 11, 7-60.

Collette B.B. & Parin N.V. 1986. Belonidae. In: Fishes of the north-eastern Atlantic and the Mediterranean (P.J.P. Whitehead, M.-L. Bauchot, J.-C. Hureau, J. Nielsen & E. Tortonese eds), pp. 604-609. UNESCO: Paris

Collette, B.B. & Parin, N.V. (1990). Belonidae. In: J.C. Quero, J.C. Hureau, C. Karrer, A. Post and L. Saldanha (eds), Check-list of the fishes of the eastern tropical Atlantic (CLOFETA), pp. 592-597. UNESCO, Paris.

Collette, B. B., & Branford, H. M. (2001). Status of the eastern Pacific agujon needlefish Tylosurus pacificusz (Steindachner, 1876)(Beloniformes: Belonidae). Revista de Biología Tropical, 49(S1), 51-57.

D'Iglio, C., Albano, M., Famulari, S., Carnevale, A., Savoca, S., Spanò, N., & Capillo, G. (2024). Intra-population variability of the saccular, utricular and lagenar otoliths of the garfish Belone belone (Linnaeus, 1760) from South-Western Ionian Sea (Central Mediterranean Sea). BMC Ecology and Evolution, 24(1), 31.

Dalyan, C., Eryılmaz, L. (2006). Two new fish records from Turkish coast of the Eastern Mediterranean: The garfish, Belone svetovidovi Collette and Parin, 1970; the spiny gurnard, Lepidotrigla dieuzeidei Audoin in Blanc and Hureau, 1973. J. Black Sea/Mediterranean Environment. 12, 155-158.

Daniel Golani & Yaniv Levy (2005). New records and rare occurrences of fish species from the Mediterranean coast of Israel, Zoology in the Middle East, 36(1), 27-32.

Dieuzeide, R., Novella, M. & Roland, J. (1954). Belonidae. In: Catalogue des poissons des Côtes Algériennes (III – Ostéptérygiens). Bull Trav Publ Stat Agricult Peche Castiglione, 2: 109-111.

Dorman, J. A. (1987). Belone svetovidovi Collette and Parin: a species of garfish new to northern Europe. Journal of the Marine Biological Association of the United Kingdom, 67(3), 679-685.

Eschmeyer W.N., Fricke R., van der Laan R. (eds.) (2024). Catalog of fishes: Genera, species, references. California Academy of Sciences, San Francisco, USA. [http:// researcharchive.calacademy.org/research/ichthyology/ catalog/fishcatmain.asp

Excoffier, L. & Smouse, P. E. (1994). Using allele frequencies and geographic subdivision to reconstruct gene trees within a species: molecular variance parsimony. Genetics, 136 (1), 343-359.

Fricke, R., Eschmeyer, W. N. & Van der Laan, R. (eds) 2024. Eschmeyer's Catalog of Fishes: Genera, Species, References. Electronic version accessed 12 May 2024.

Froese, R., Pauly, D. (2024). FishBase. Available at: http://www. fishbase.org, Version (02 ⁄ 2024).

Ghailen, H., Abdallah, H., Hassan, A., Mourad, C., Abderrahmen, B., & Othman, J. (2010). Length-weight relationships for 13 fish species from the Gulf of Gabes (Southern Tunisia, Central Mediterranean). African Journal of Biotechnology, 9 (37), 6177-6181.

Golani, D., Ozturk, B., Basusta, N. (2006). Fishes of the eastern Mediterranean. Turkish Marine Research Foundation. Publication No. 24, Istanbul, Turkey, 259 pp. ISBN 975 8825-12-7.

Hall, T. A. (1999). BioEdit: a user-friendly biological sequence alignment editor and analysis program for Windows 95/98/NT. Nucleic. Acids. Symp. Ser., 41(41): 95-98.

Hall, B. G. (2013). Building phylogenetic trees from molecular data with MEGA. Molecular biology and evolution, 30(5), 1229-1235.

Imsiridou, A., Minos, G., Kokokiris, L., Alexandrou, M., Kyriakidou, M., & Karidas, T. (2016). Genetic and phenotypic identification of Tylosurus acus imperialis in Thermaikos Gulf, North Aegean Sea. Cahiers de Biologie Marine, 57, 9-15.

Kalayci, F., & Yeşilçiçek, T. (2012). Length based seasonal growth of the garfish, Belone belone (Linnaeus, 1761)(Belonidae), in the southeast Black Sea. African Journal of Biotechnology, 11(35), 8742-8750.

Kara, A., Sağlam, C., Acarli, D., & Cengız, Ö. (2018). Length-weight relationships for 48 fish species of the Gediz estuary, in Izmir Bay (Central Aegean Sea, Turkey). Journal of the Marine Biological Association of the United Kingdom, 98(4), 879-884.

Karachle, P. K., & Stergiou, K. I. (2008). Length–length and length–weight relationships of several fish species from the North Aegean Sea (Greece). Journal of Biological Research-Thessaloniki, 10, 149-157.

Karataş, A., Filiz, H., Erciyas-Yavuz, K., Özeren, S. C., & Tok, C. V. (2021). The vertebrate biodiversity of Turkey. In Biodiversity, Conservation and Sustainability in Asia: Volume 1: Prospects and Challenges in West Asia and Caucasus (pp. 175-274). Cham: Springer International Publishing.

Keskin, Ç., & Gaygusuz, Ö. (2010). Length-weight relationships of fishes in shallow waters of Erdek Bay (Sea of Marmara, Turkey). European Journal of Biology, 69(2), 87-94.

Kocher, T. D., Thomas, W. K., Meyer, A., Edwards, S. V., Pääbo, S., Villablanca, F. X., & Wilson, A. C. (1989). Dynamics of mitochondrial DNA evolution in animals: amplification and sequencing with conserved primers. Proceedings of the National Academy of Sciences, 86(16), 6196-6200.

Kumar, S., G. Stecher, Li, M., Knyaz C. and Tamura, K. (2018). MEGA X: Molecular evolutionary genetics analysis across computing platforms. Mol. Biol. Evol., 35(6): 1547.

Kutlu, Y., & Turan, C. (2018). An Intelligent Software for Measurements of Biological Materials: BioMorph. Natural and Engineering Sciences, 3(2), 225-233.

Leigh, J. W., Bryant, D., & Nakagawa, S. (2015). POPART: full-feature software for haplotype network construction. Methods in Ecology & Evolution, 6(9).

Lovejoy, N. R. (2000). Reinterpreting recapitulation: systematics of needlefishes and their allies (Teleostei: Beloniformes). Evolution, 54(4), 1349-1362.

Meriç, N., Altun, Ö. (1999). The garfish, Belone svetovidovi Collette and Parin, 1970, new to the Aegean Sea. Israel J. Zoology 45, 423-426.

Muus, B. J., & Nielsen, J. G. (1999). Sea fish. Scandinavian fishing year book. Hedehusene, Denmark. 340 p. DOI/87-90787-00-5.

Near, T. J., Dornburg, A., Eytan, R. I., Keck, B. P., Smith, W. L., Kuhn, K. L., ... & Wainwright, P. C. (2013). Phylogeny and tempo of diversification in the

superradiation of spiny-rayed fishes. Proceedings of the national Academy of sciences, 110(31), 12738-12743.

Nei, M. (1987). Molecular Evolutionary Genetics. Columbia University Press, New York

Nelson, J. S., Grande, T. C., & Wilson, M. V. (2016). Fishes of the World. John Wiley & Sons.

Öztürk, D. S. (2023). Molecular and Phenotypic Characteristics of Short-Beaked Garfish Belone svetovidovi Collette and Parin, 1970 in a New Location, the Sea of Marmara. Marine Science and Technology Bulletin, 12(2), 156-161.

Parin N.V. (1967). Review of the marine Belonidae of the western Pasific and Indian Ocean. Trduy Institute Okeanologil, 84, 2-83.

Paugy, D. 2003. Belonidae. p. 293-297. In C. Lévêque, D. Paugy and G.G. Teugels (eds.) Faune des poissons d'eaux douce et saumâtres de l'Afrique de l'Ouest, Tome 2. Coll. Faune et Flore tropicales 40. Musée Royal de l'Afrique Centrale, Tervuren, Belgique, Museum National d'Histoire Naturalle, Paris, France and Institut de Recherche pour le Développement, Paris, France. 815 p.

Polat, N., İnceismail, Y., Yılmaz, S., & Bostancı, D. (2009). Age determination, age-length and length-weight relationships of garfish (Belone belone L., 1761) in the Black Sea (Samsun).

Sambrook, J., Fritsch, E. F., & Maniatis, T. (1989). Molecular cloning: a laboratory manual (No. Ed. 2). Cold spring harbor laboratory press.

Samsun, S., & Sağlam, N. E. (2021). Length-weight relationships and condition factors of six fish species in the southern black sea (Ordu-Turkey). Journal of Agricultural Faculty of Gaziosmanpaşa University (JAFAG), 38(2), 111-116.

Samsun, S., & Sağlam, N. E. (2024). Determination of Growth and Reproduction Characteristics of Garfish (*Belone euxini* Günther, 1866) in the South Black Sea. Thalassas: An International Journal of Marine Sciences, 1-11.

Samsun, O., Samsun, N., Bilgin, S., & Kalayci, F. (2006). Population biology and status of exploitation of introduced garfish *Belone belone euxini* (Günther, 1866) in the Black Sea. Journal of Applied Ichthyology, 22(5), 353-356.

Samsun, O., Akyol, O., Ceyhan, T., & Erdem, Y. (2017). Length-weight relationships for 11 fish species from the Central Black Sea, Turkey. Ege Journal of Fisheries and Aquatic Sciences, 34(4), 455-458.

Sanger, F., Nicklen, S. and Coulson. A. R. (1977). DNA sequencing with chain-terminating inhibitors. Proc. Natl. Acad. Sci., 74(12): 5463-5467.

Schneider, S., Roessli, D. & Excoffier, L. (2000). Arlequin: a software for population genetics data analysis. User Manual Ver, 2, 2496-2497.

Sinis, A.I. (2005). First record of Tylosurus crocodilus (Peron & Lesueur 1821) (Pisces: Belonidae) in the Mediterranean (North Aegean Sea, Greece). Journal of Biological Research (Thessaloniki), 4, 221-224.

Tajima, F. (1983). Evolutionary relationship of DNA sequences in finite populations. Genetics, 105 (2), 437-460.

Thompson, J. D., Higgins, D.G., Gibson, T.J. (1994). CLUSTAL W: improving the sensitivity of progressive multiple sequence alignment through sequence weighting, position-specific gap penalties and weight matrix choice. Nucleic Acids Research, 22: 4673-4680.

Toji, T., Abdussamad, E. M., Ameri, S., Sijad, B., & Sajikumar, K. K. (2024). An integrative taxonomic study on needlefishes (Belonidae) unveils two new species within the genus Ablennes from the Indian Ocean. Regional Studies in Marine Science, 103522.

Turan, C., Yağlıoğlu, D., Doğdu, S. A., Ergüden, D., Ivanova, P. P., & Raykov, V. S. (2023). Existence of *Belone svetovidovi* Collette & Parin, 1970 (Family: Belonidae) in the Marmara Sea and Black Sea Coasts of Türkiye. Natural and Engineering Sciences, 8(2), 72-81.

Uçkun, D., Akalin, S., Taşkavak, E., & Toğulga, M. (2004). Some biological characteristics of the garfish (Belone belone L., 1761) in Izmir Bay, Aegean Sea. Journal of Applied Ichthyology, 20(5), 413-416.

Ward, R. D., Zemlak, T. S., Innes, B. H., Last P. R. and Hebert, P. D. (2005). DNA barcoding Australia's fish species. Philos. Trans. R. Soc. Lond., B, Biol. Sci., 360(1462): 1847-1857.

Yankova, M., Raykov, V., Ivanova, P., Dzhembekova, N., Turan, C., & Raev, Y. (2023). Morphological and genetic characteristics of garfish *Belone belone* (L., 1760) (Belonidae, Teleostei) population from the southern Bulgarian Black Sea coast. Nature Conservation, 54, 1.

Zorica, B., & Keč, V. Č. (2013). Age, growth and mortality of the garfish, Belone belone (L. 1761) in the Adriatic Sea. Journal of the Marine Biological Association of the United Kingdom, 93(2), 365-372.

Zorica, B., Sinovčić, G., & Keč, V. Č. (2011). The reproductive cycle, size at maturity and fecundity of garfish (Belone belone, L. 1761) in the eastern Adriatic Sea. Helgoland Marine Research, 65, 435-444.

Klingenberg, C. P. (1996). Multivariate allometry. In Advances in morphometrics (pp. 23-49). Boston, MA: Springer US.

Jolicoeur, P. 1963. The multivariate generalization of the allometry equation. Biometrics 19:497499.

Figure 1

Click here to access/download;Figure;Fig 1.tiff

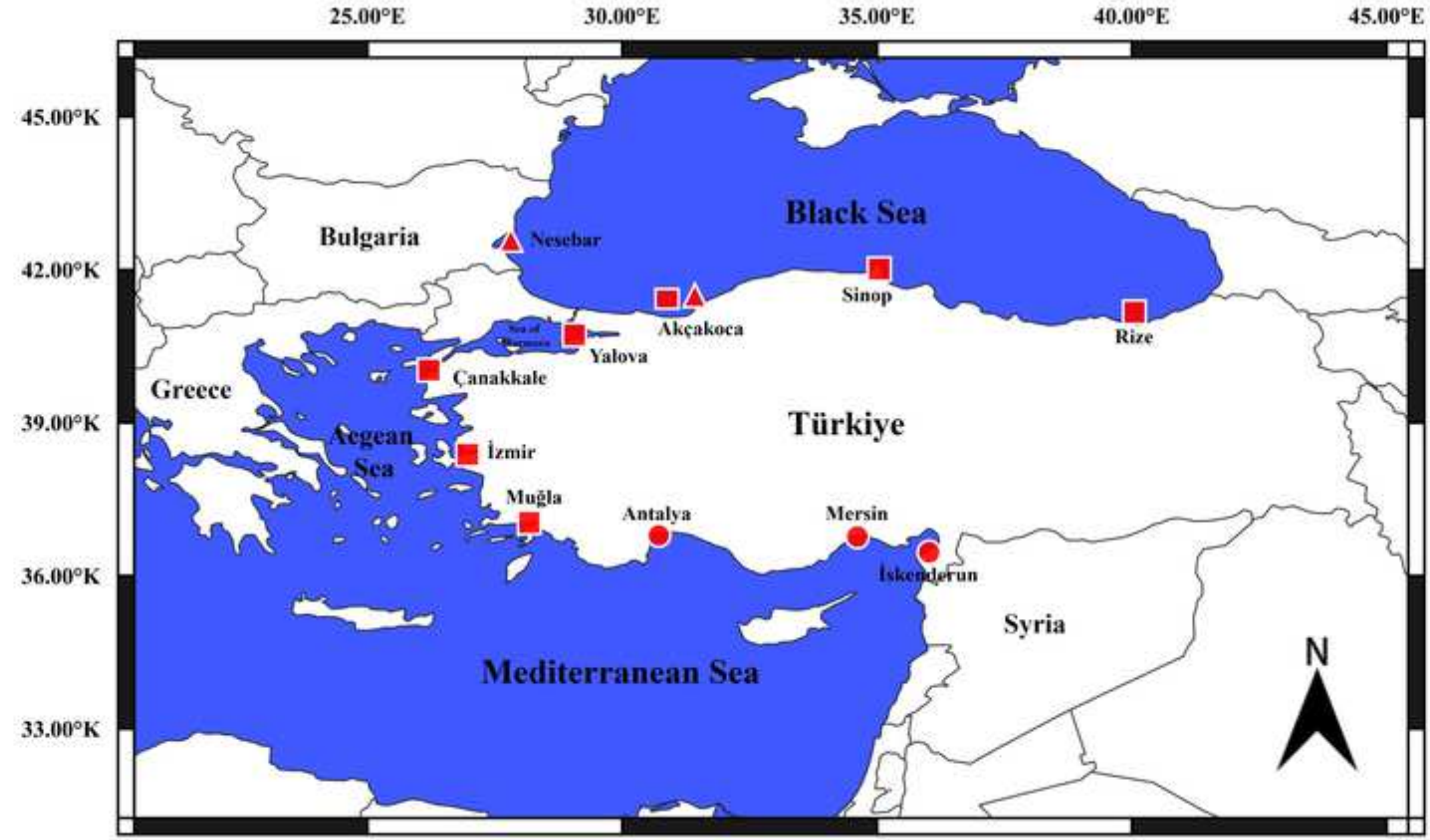

Figure 2

Click here to access/download;Figure;Fig 2.tiff

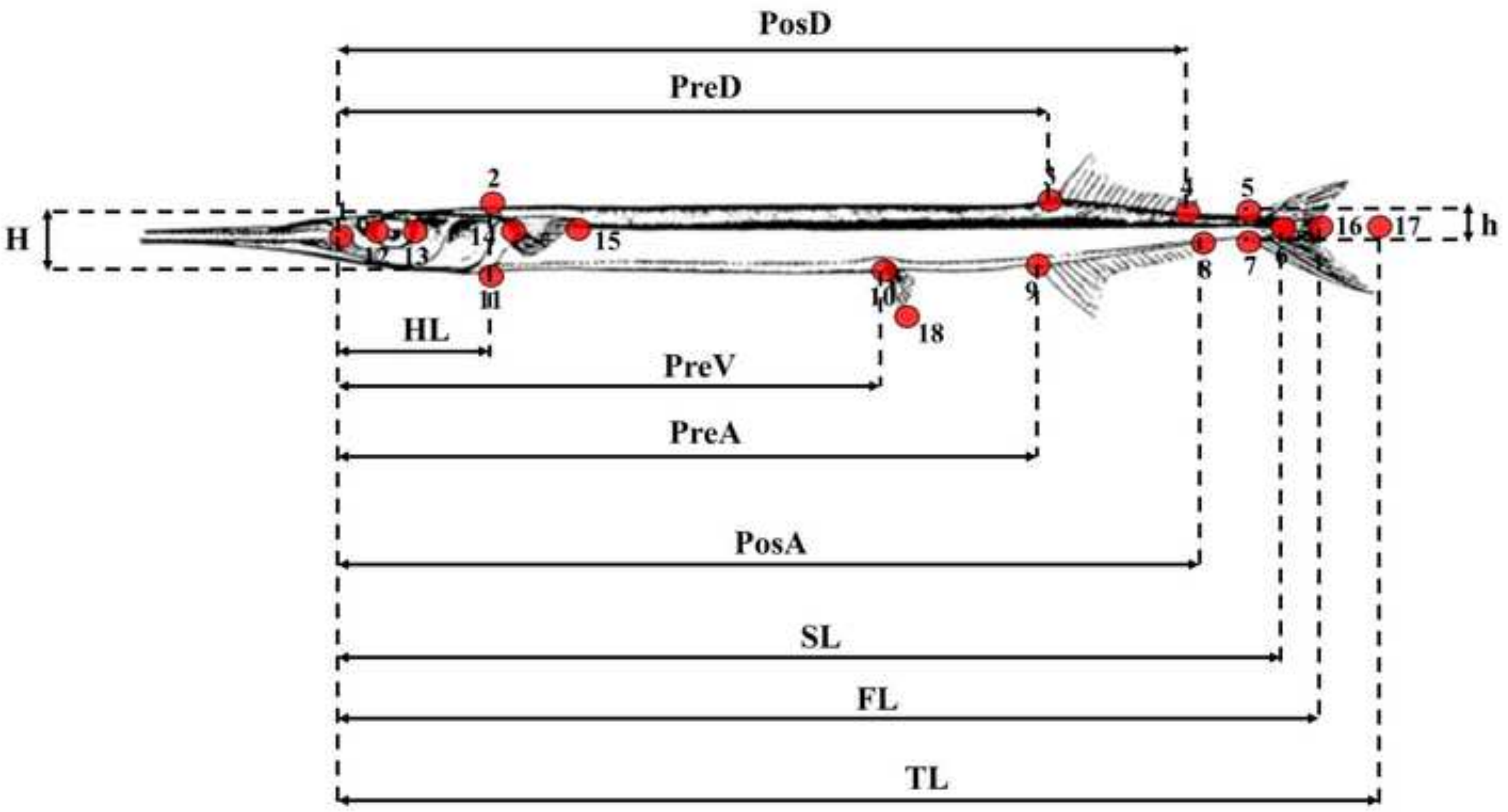

Figure 3

Click here to access/download;Figure;Fig 3.tiff

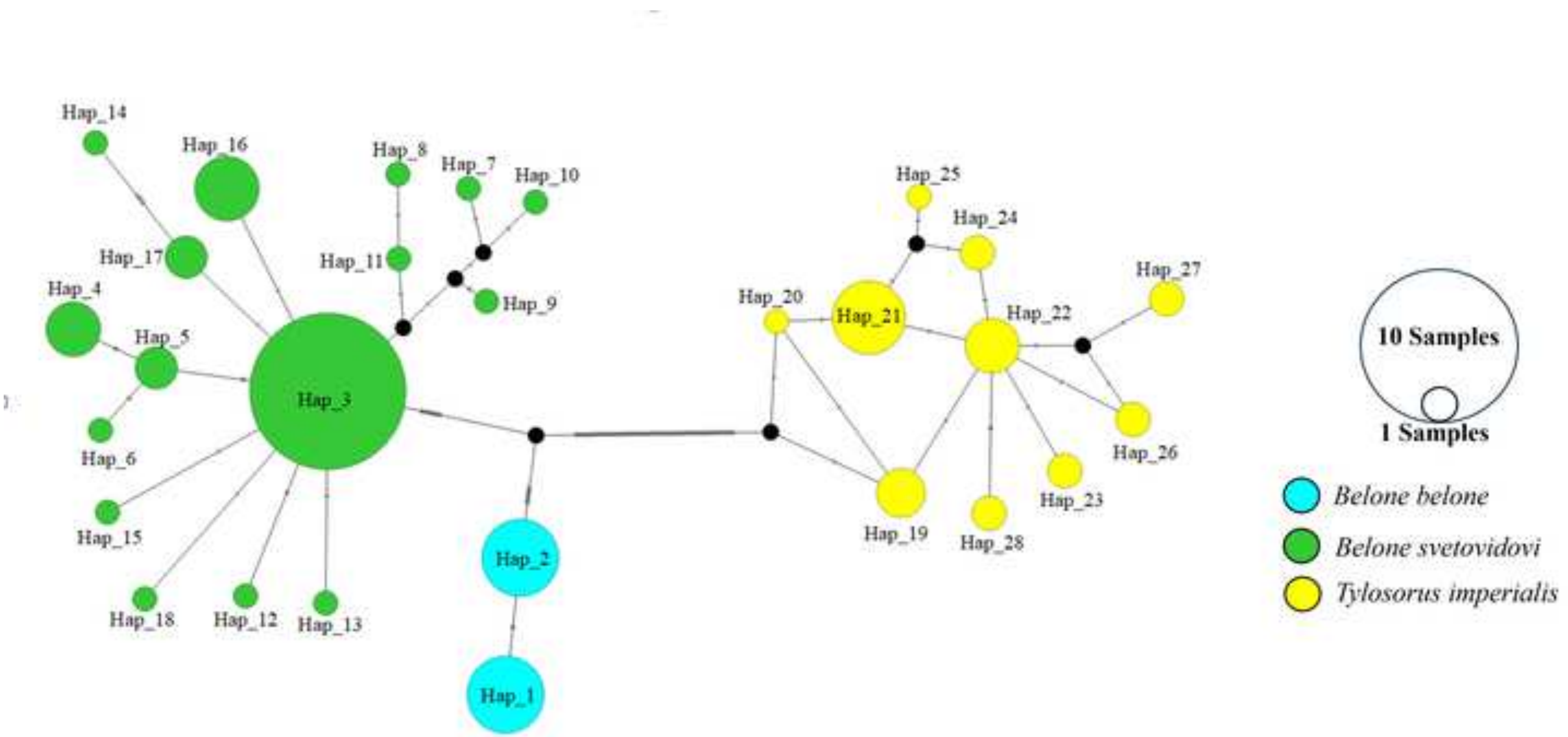

Figure 4

Click here to access/download;Figure;Fig 4.tif

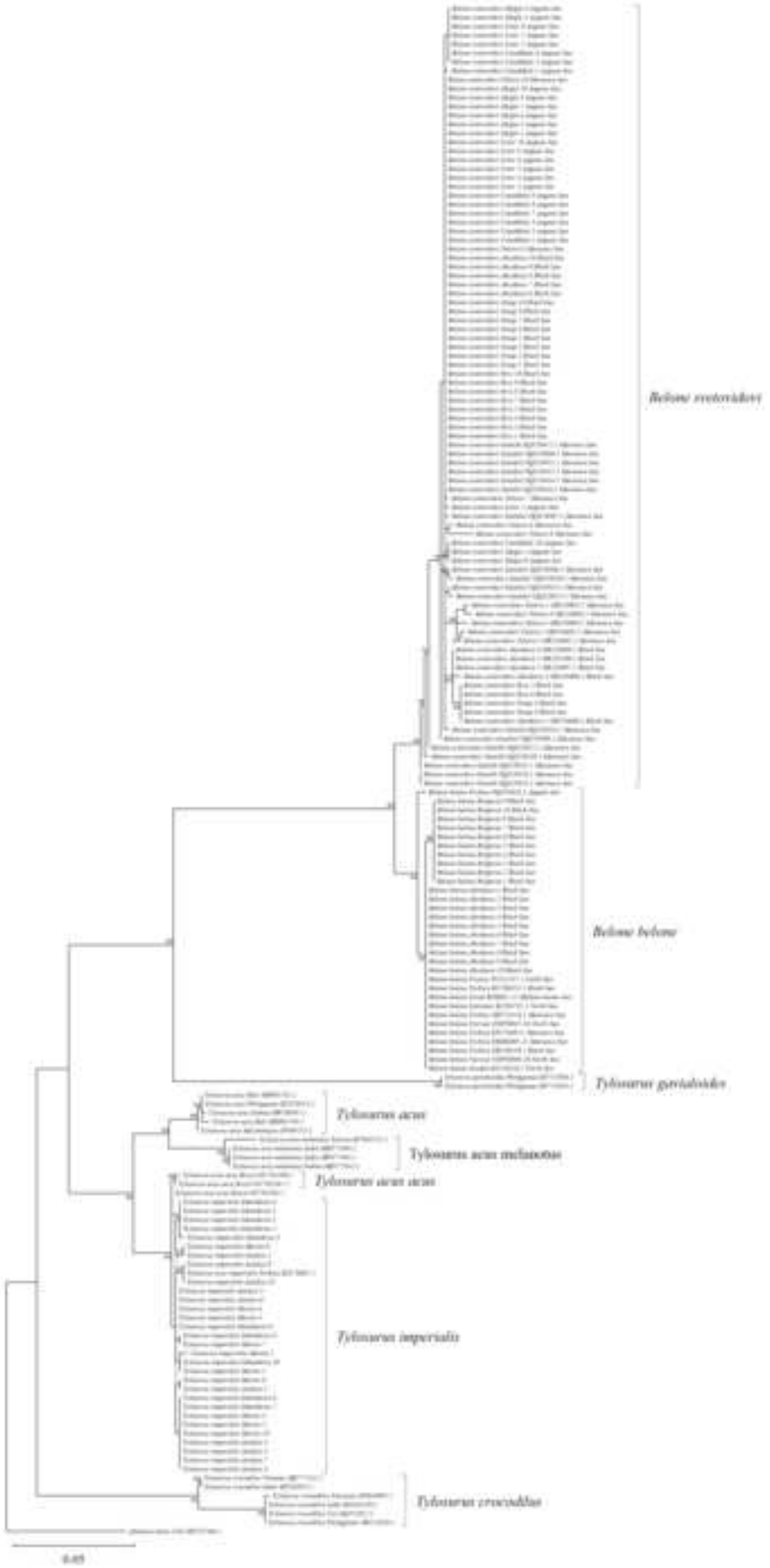

Figure 5 Click here to access/download;Figure;Fig 5.tif

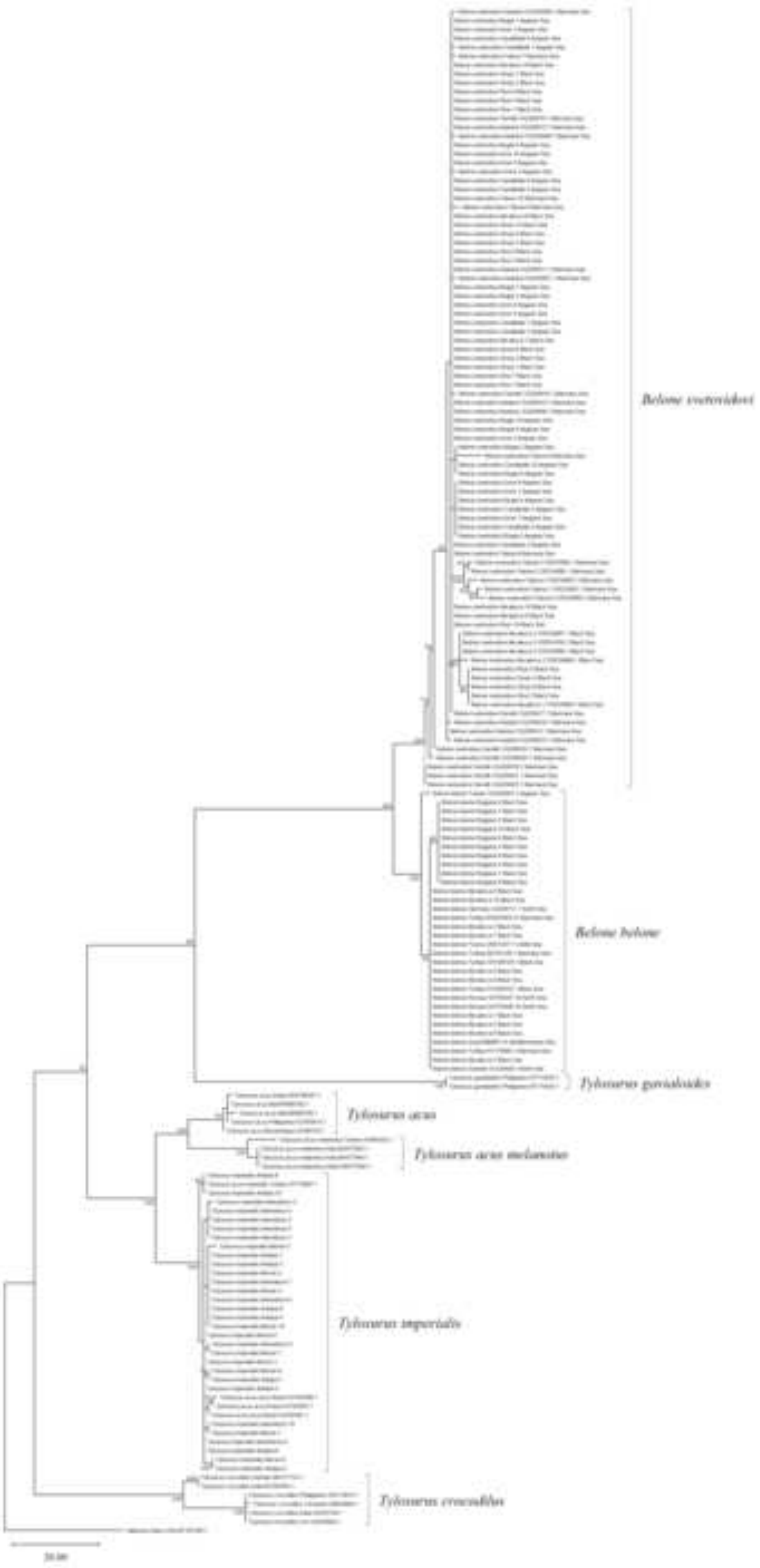

Figure 6

Click here to access/download;Figure;Fig 6.tiff

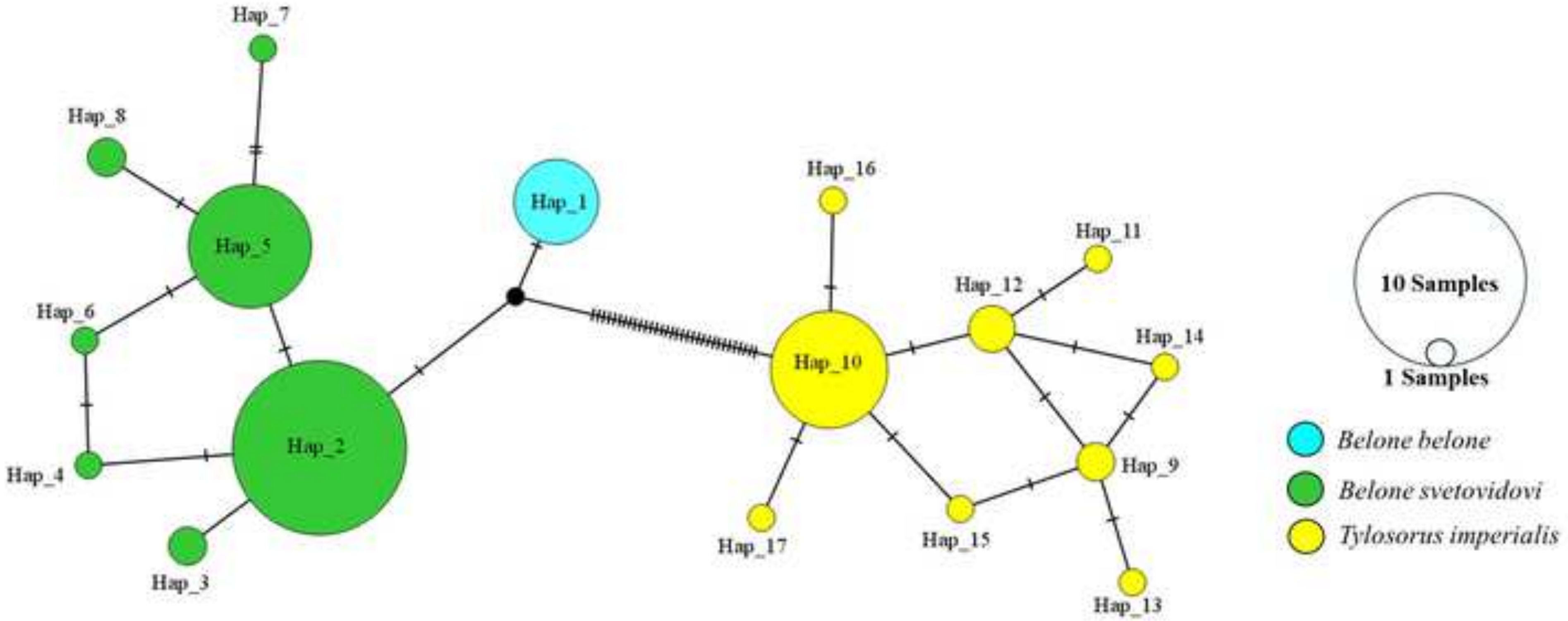

Click here to access/download;Figure;Fig 7.tiff

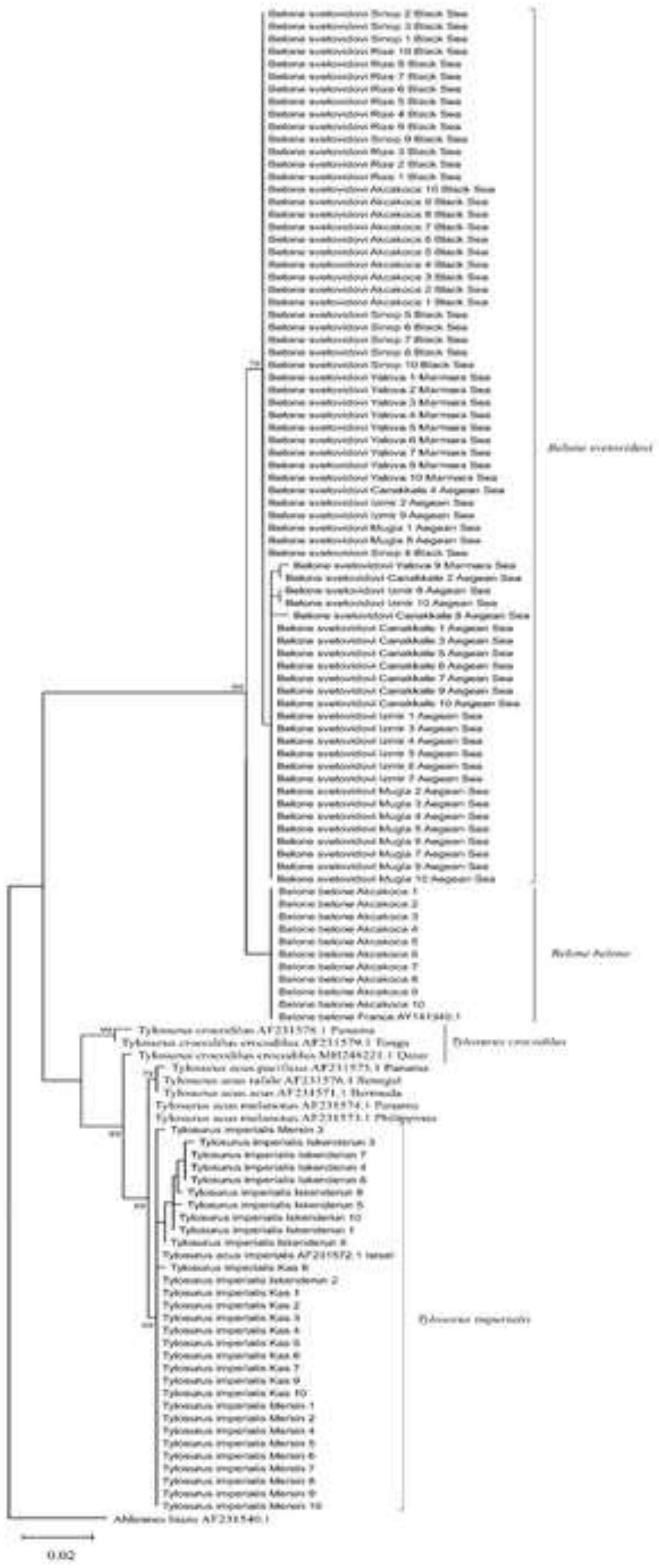

Figure 8

Click here to access/download;Figure;Fig 8.tif

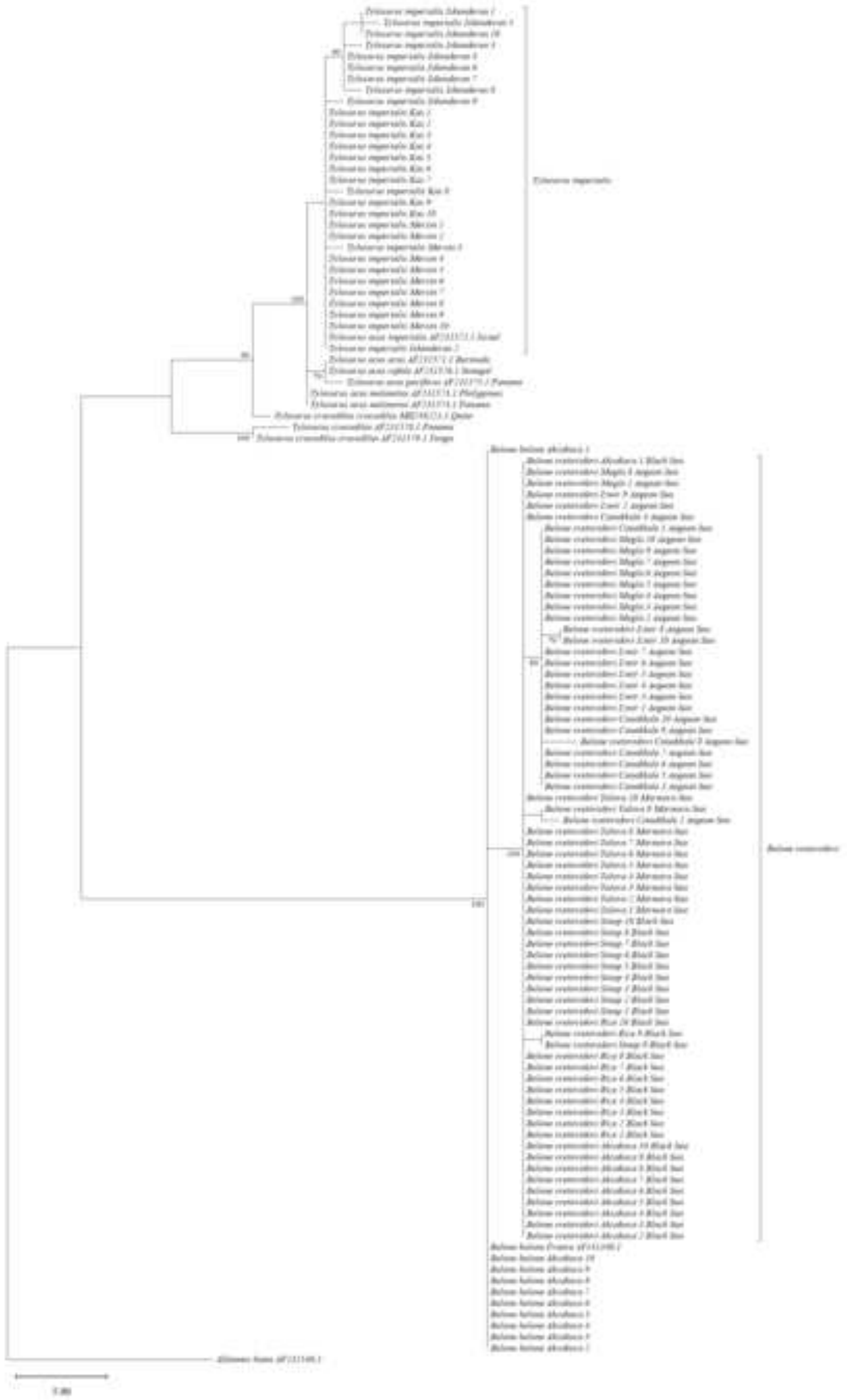

Figure 9

Click here to access/download;Figure;Fig 9.tiff

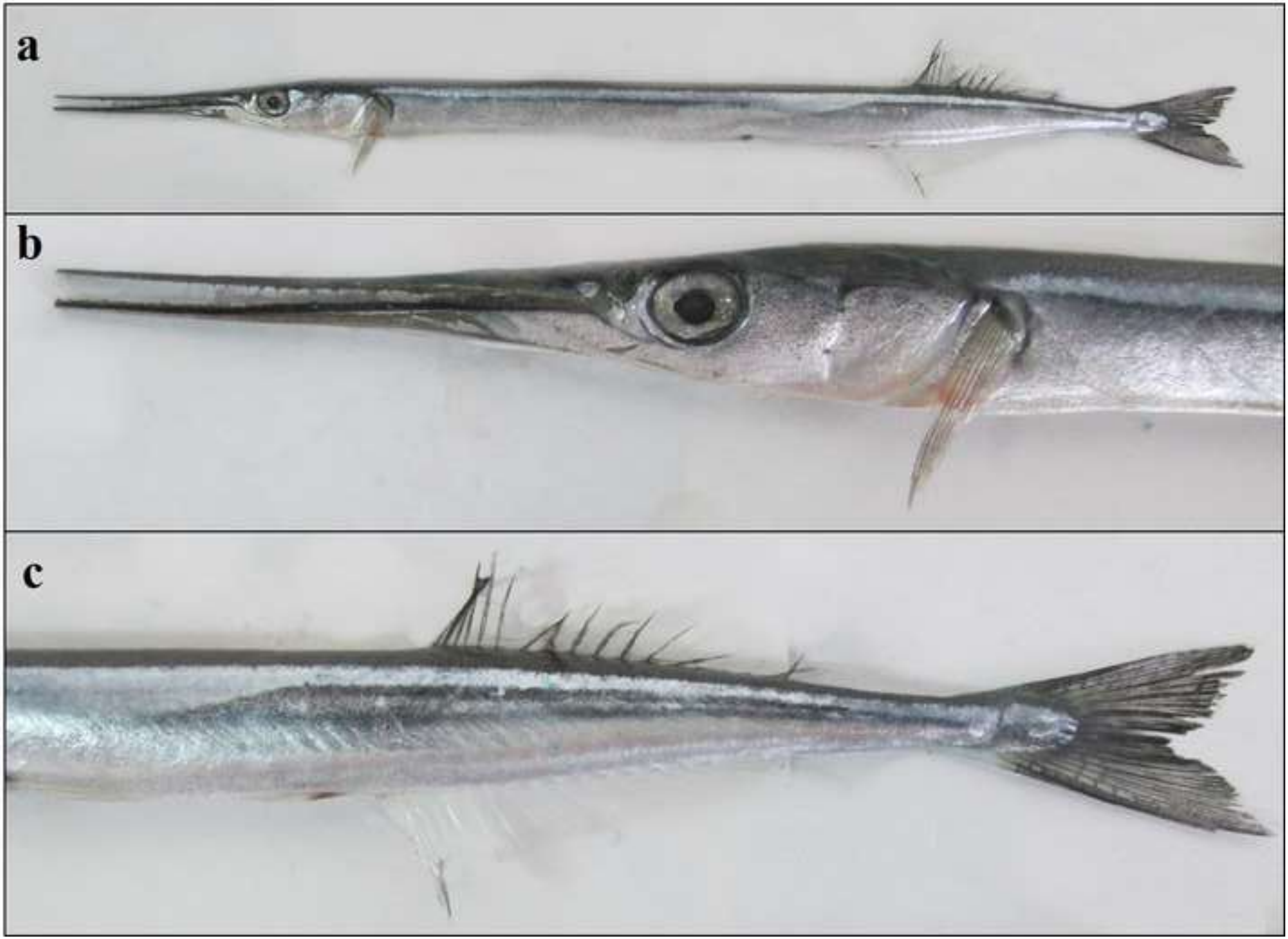

Figure 10

Click here to access/download;Figure;Fig 10.tiff

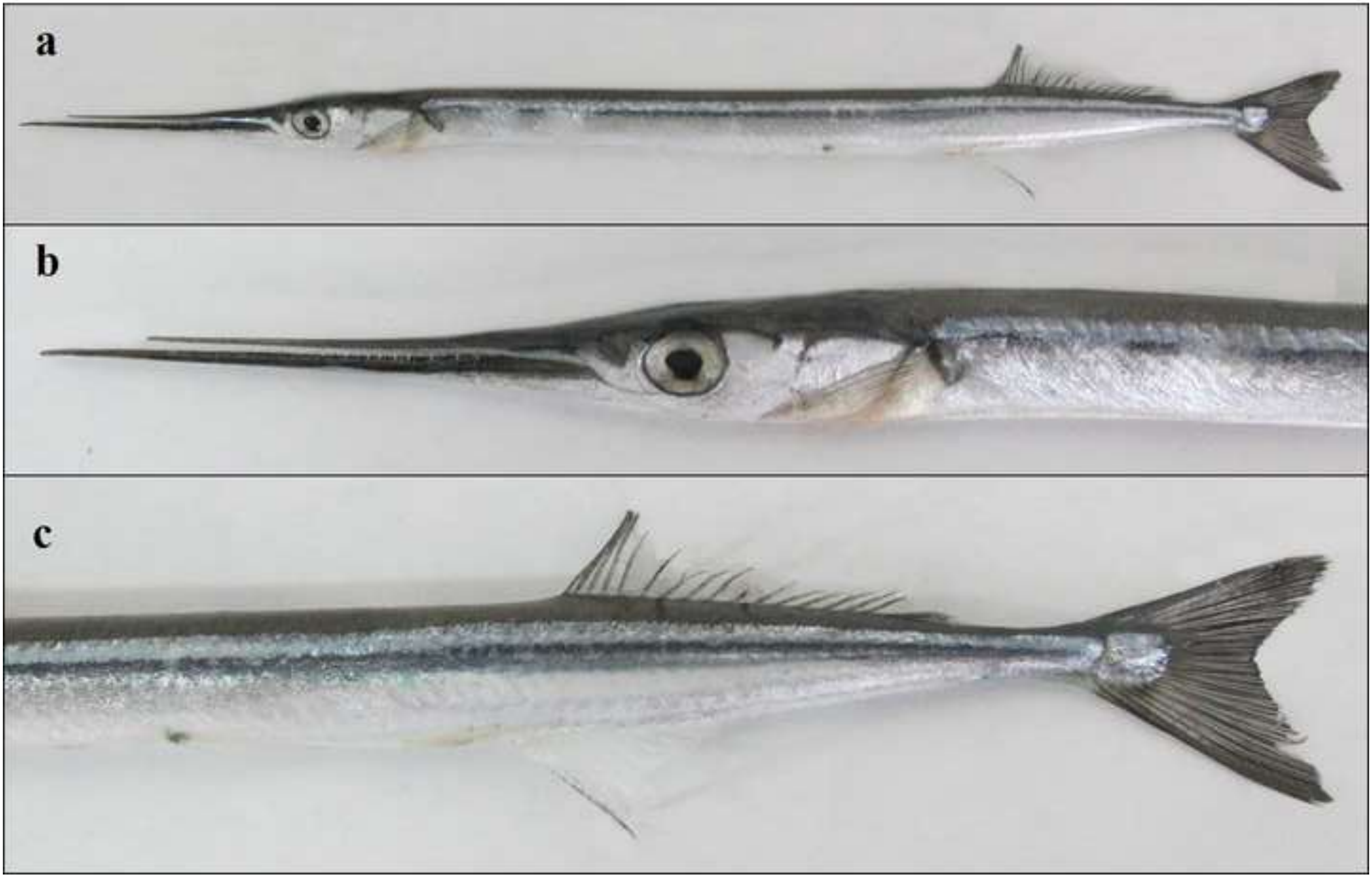

Figure 11

Click here to access/download;Figure;Fig 11.tiff

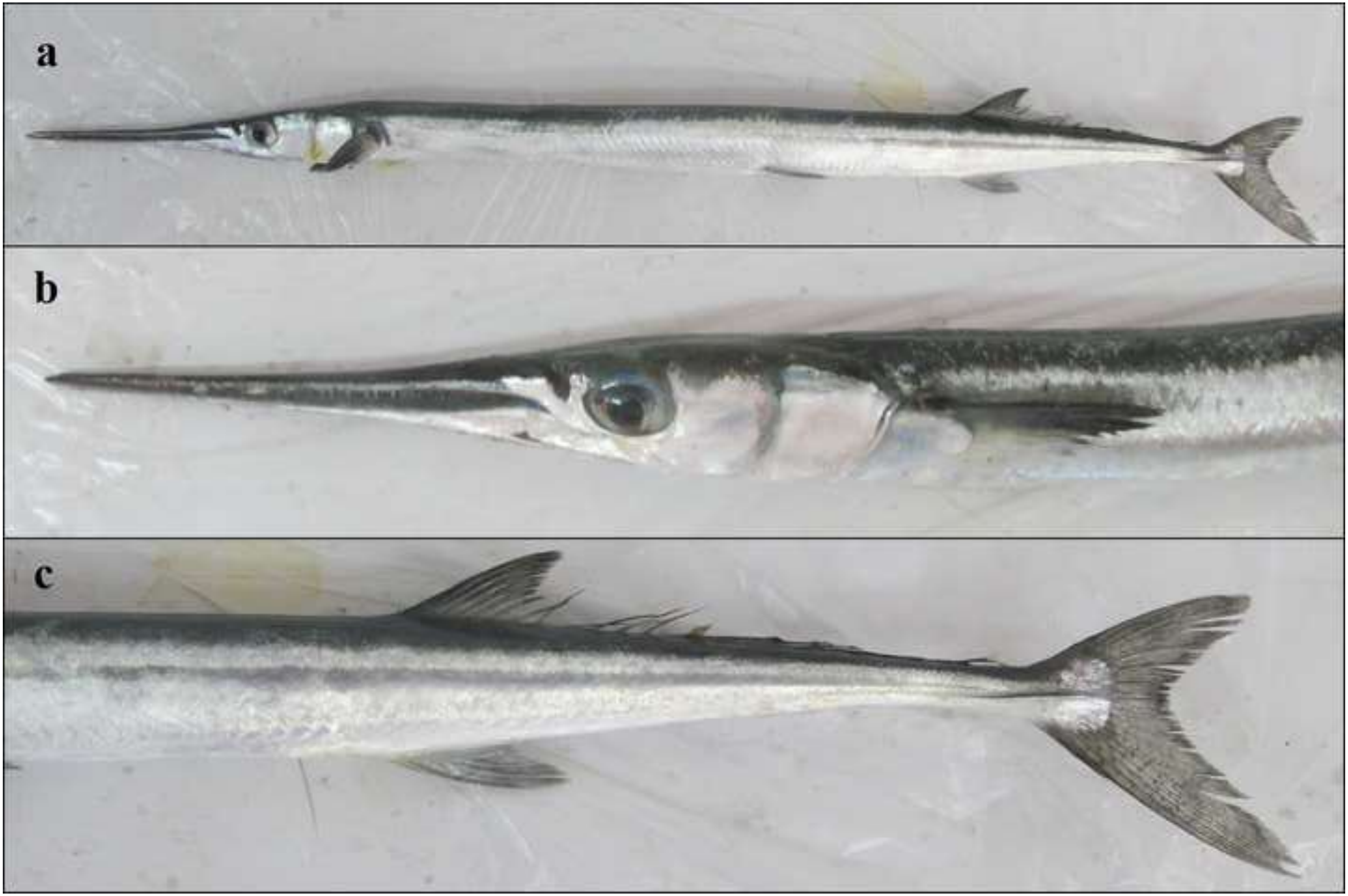

Figure 12

Click here to access/download;Figure;Fig 12.tiff

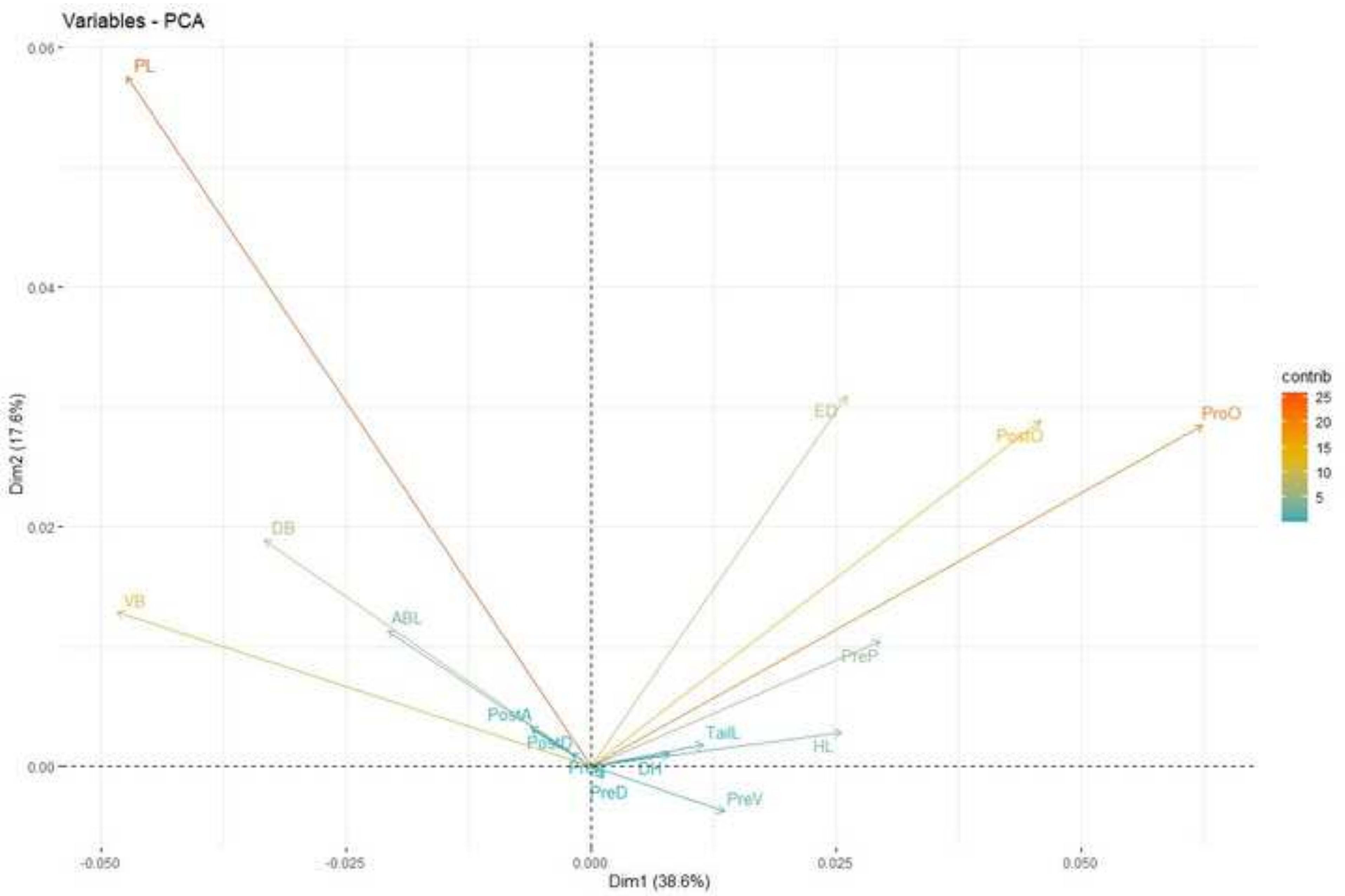

Figure 13

Click here to access/download;Figure;Fig 13.tiff

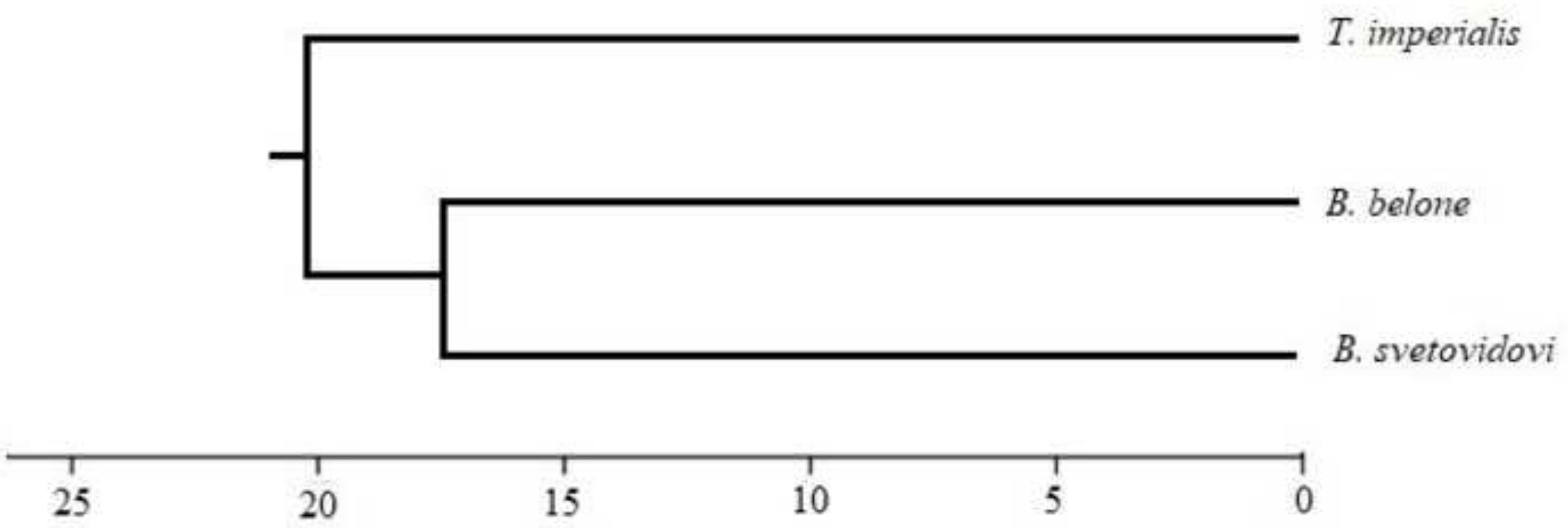

Click here to access/download

Supporting Information

Appendix 1.pdf
