# Supplementary material for: Systematic identification of needlefish (Belonidae) species using molecular genetic and morphological markers in the Mediterranean and Black Seas
Source: PLoS One. 2025 Feb 28;20(2):e0315401. doi: 10.1371/journal.pone.0315401 (PMC11870352; doi:10.1371/journal.pone.0315401)
Supplement: Attachment — Submitted filename: Response to Reviewers.docx [file pone.0315401.s006.docx]

**Journal requirements and Response to Reviewers**

1. Please ensure that your manuscript meets PLOS ONE's style requirements, including those for file naming. The PLOS ONE style templates can be found at

Action: Necessary style adjustments have been made.

2. We noticed you have some minor occurrence of overlapping text with the following previous publication(s), which needs to be addressed:

- Morphological and genetic characteristics of garfish Belone belone (L., 1760) (Belonidae, Teleostei) population from the southern Bulgarian Black Sea coast (DOI:10.3897/natureconservation.54.113071)

-Family Belonidae Bonaparte 1832 (https://www.calacademy.org/sites/default/files/assets/docs/belonidae.pdf)

-Existence of Belone svetovidovi Collette & Parin, 1970 in the Marmara Sea and Black Sea Coasts of Türkiye (doi: 10.28978/nesciences.1331296)

(among others)

In your revision ensure you cite all your sources (including your own works), and quote or rephrase any duplicated text outside the methods section. Further consideration is dependent on these concerns being addressed.

Action: The necessary arrangements have been made as commented.

3. In your Methods section, please provide additional information regarding the permits you obtained for the work. Please ensure you have included the full name of the authority that approved the field site access and, if no permits were required, a brief statement explaining why.

Action: The necessary information has been added to the relevant section as suggested.

4. We suggest you thoroughly copyedit your manuscript for language usage, spelling, and grammar. If you do not know anyone who can help you do this, you may wish to consider employing a professional scientific editing service.

The American Journal Experts (AJE) (https://www.aje.com/) is one such service that has extensive experience helping authors meet PLOS guidelines and can provide language editing, translation, manuscript formatting, and figure formatting to ensure your manuscript meets our submission guidelines. Please note that having the manuscript copyedited by AJE or any other editing services does not guarantee selection for peer review or acceptance for publication.

Upon resubmission, please provide the following:

The name of the colleague or the details of the professional service that edited your manuscript

A copy of your manuscript showing your changes by either highlighting them or using track changes (uploaded as a *supporting information* file)

A clean copy of the edited manuscript (uploaded as the new *manuscript* file)

Action: Language editing was received from Prof. Dr. Alen Soldo whose first name is English.

Action: The manuscript was thoroughly checked for language usage, spelling, and grammar.

5. Thank you for stating the following financial disclosure:

“This research was supported by the Scientific & Technological Research Council of Turkey (TUBITAK-121N777) and Bulgarian Academy of Sciences (BAS-IC-TR/1/2022-2023). The authors thank to TUBITAK for their supports.”

At this time, please address the following queries:

a) Please clarify the sources of funding (financial or material support) for your study. List the grants or organizations that supported your study, including funding received from your institution.

Action:

5.a- This research was supported by the Scientific & Technological Research Council of Türkiye-2502 programe

b) State what role the funders took in the study. If the funders had no role in your study, please state: “The funders had no role in study design, data collection and analysis, decision to publish, or preparation of the manuscript.”

Action:

5.b-The funders had no role in study design, data collection and analysis, decision to publish, or preparation of the manuscript

c) If any authors received a salary from any of your funders, please state which authors and which funders.

Action:

5.c- No

d) If you did not receive any funding for this study, please state: “The authors received no specific funding for this work.”

Action:

5.d- The authors received no specific funding for this work

Please include your amended statements within your cover letter; we will change the online submission form on your behalf.

6. Thank you for stating the following in the Acknowledgments Section of your manuscript:

“This research was supported by the Scientific & Technological Research Council of Turkey (TUBITAK-121N777) and Bulgarian Academy of Sciences (BAS-IC-TR/1/2022-2023). The authors thank to TUBITAK for their supports.”

We note that you have provided funding information that is currently declared in your Funding Statement. However, funding information should not appear in the Acknowledgments section or other areas of your manuscript. We will only publish funding information present in the Funding Statement section of the online submission form.

Please remove any funding-related text from the manuscript and let us know how you would like to update your Funding Statement. Currently, your Funding Statement reads as follows:

“This research was supported by the Scientific & Technological Research Council of Turkey (TUBITAK-121N777) and Bulgarian Academy of Sciences (BAS-IC-TR/1/2022-2023). The authors thank to TUBITAK for their supports.”

Please include your amended statements within your cover letter; we will change the online submission form on your behalf.

Action: Funding information was presented in the Funding Statement section of the online submission form

7. In the online submission form you indicate that your data is not available for proprietary reasons and have provided a contact point for accessing this data. Please note that your current contact point is a co-author on this manuscript. According to our Data Policy, the contact point must not be an author on the manuscript and must be an institutional contact, ideally not an individual. Please revise your data statement to a non-author institutional point of contact, such as a data access or ethics committee, and send this to us via return email. Please also include contact information for the third party organization, and please include the full citation of where the data can be found.

Action: In the online submission form, we chose this section by mistake, now, it has been corrected as “The data are all contained within the manuscript and/or Supporting Information files”. All sequence files are available from the NCBI (GenBank) database (accession numbers are given in the manuscript). All relevant morfological data are within the Supporting Information files.

8. We note that Figures 2, 9, 10 and 11 in your submission contain copyrighted images. All PLOS content is published under the Creative Commons Attribution License (CC BY 4.0), which means that the manuscript, images, and Supporting Information files will be freely available online, and any third party is permitted to access, download, copy, distribute, and use these materials in any way, even commercially, with proper attribution. For more information, see our copyright guidelines: <http://journals.plos.org/plosone/s/licenses-and-copyright>.

We require you to either (1) present written permission from the copyright holder to publish these figures specifically under the CC BY 4.0 license, or (2) remove the figures from your submission:

1. You may seek permission from the original copyright holder of Figures 2, 9, 10 and 11 to publish the content specifically under the CC BY 4.0 license.

We recommend that you contact the original copyright holder with the Content Permission Form (<http://journals.plos.org/plosone/s/file?id=7c09/content-permission-form.pdf>) and the following text:

“I request permission for the open-access journal PLOS ONE to publish XXX under the Creative Commons Attribution License (CCAL) CC BY 4.0 (<http://creativecommons.org/licenses/by/4.0/>). Please be aware that this license allows unrestricted use and distribution, even commercially, by third parties. Please reply and provide explicit written permission to publish XXX under a CC BY license and complete the attached form.”

Please upload the completed Content Permission Form or other proof of granted permissions as an "Other" file with your submission.

In the figure caption of the copyrighted figure, please include the following text: “Reprinted from [ref] under a CC BY license, with permission from [name of publisher], original copyright [original copyright year].”

2. If you are unable to obtain permission from the original copyright holder to publish these figures under the CC BY 4.0 license or if the copyright holder’s requirements are incompatible with the CC BY 4.0 license, please either i) remove the figure or ii) supply a replacement figure that complies with the CC BY 4.0 license. Please check copyright information on all replacement figures and update the figure caption with source information. If applicable, please specify in the figure caption text when a figure is similar but not identical to the original image and is therefore for illustrative purposes only.

Action: Figures 9, 10 and 11 were created from the photographs taken by us within the scope of our study. Figure 2 is taken from a scientific article that is open to public use and edited according to our article and the necessary reference is made in the figure description.

9. We note that Figure 1 in your submission contain [map/satellite] images which may be copyrighted. All PLOS content is published under the Creative Commons Attribution License (CC BY 4.0), which means that the manuscript, images, and Supporting Information files will be freely available online, and any third party is permitted to access, download, copy, distribute, and use these materials in any way, even commercially, with proper attribution. For these reasons, we cannot publish previously copyrighted maps or satellite images created using proprietary data, such as Google software (Google Maps, Street View, and Earth). For more information, see our copyright guidelines: http://journals.plos.org/plosone/s/licenses-and-copyright.

We require you to either (1) present written permission from the copyright holder to publish these figures specifically under the CC BY 4.0 license, or (2) remove the figures from your submission:

1. You may seek permission from the original copyright holder of Figure 1 to publish the content specifically under the CC BY 4.0 license.

We recommend that you contact the original copyright holder with the Content Permission Form (http://journals.plos.org/plosone/s/file?id=7c09/content-permission-form.pdf) and the following text:

“I request permission for the open-access journal PLOS ONE to publish XXX under the Creative Commons Attribution License (CCAL) CC BY 4.0 (http://creativecommons.org/licenses/by/4.0/). Please be aware that this license allows unrestricted use and distribution, even commercially, by third parties. Please reply and provide explicit written permission to publish XXX under a CC BY license and complete the attached form.”

Please upload the completed Content Permission Form or other proof of granted permissions as an "Other" file with your submission.

In the figure caption of the copyrighted figure, please include the following text: “Reprinted from [ref] under a CC BY license, with permission from [name of publisher], original copyright [original copyright year].”

2. If you are unable to obtain permission from the original copyright holder to publish these figures under the CC BY 4.0 license or if the copyright holder’s requirements are incompatible with the CC BY 4.0 license, please either i) remove the figure or ii) supply a replacement figure that complies with the CC BY 4.0 license. Please check copyright information on all replacement figures and update the figure caption with source information. If applicable, please specify in the figure caption text when a figure is similar but not identical to the original image and is therefore for illustrative purposes only.

The following resources for replacing copyrighted map figures may be helpful:

USGS National Map Viewer (public domain): http://viewer.nationalmap.gov/viewer/

The Gateway to Astronaut Photography of Earth (public domain): http://eol.jsc.nasa.gov/sseop/clickmap/

Maps at the CIA (public domain): https://www.cia.gov/library/publications/the-world-factbook/index.html and https://www.cia.gov/library/publications/cia-maps-publications/index.html

NASA Earth Observatory (public domain): http://earthobservatory.nasa.gov/

Landsat: http://landsat.visibleearth.nasa.gov/

USGS EROS (Earth Resources Observatory and Science (EROS) Center) (public domain): http://eros.usgs.gov/#

Natural Earth (public domain): <http://www.naturalearthdata.com/>

Action: Figure 1 was created by us in qgis programe with open source and copyright-free maps. Therefore, no copyright authorisation is required.

10. Please include captions for your Supporting Information files at the end of your manuscript, and update any in-text citations to match accordingly. Please see our Supporting Information guidelines for more information: <http://journals.plos.org/plosone/s/supporting-information>.

Action: Captions for your Supporting Information files were added at the end of manuscript, and citations in-text updated to match accordingly.

**Comments to the Author**

1. Is the manuscript technically sound, and do the data support the conclusions?

The manuscript must describe a technically sound piece of scientific research with data that supports the conclusions. Experiments must have been conducted rigorously, with appropriate controls, replication, and sample sizes. The conclusions must be drawn appropriately based on the data presented.

Reviewer #1: Partly

Reviewer #2: Yes

Reviewer #3: Partly

2. Has the statistical analysis been performed appropriately and rigorously?

Reviewer #1: N/A

Reviewer #2: Yes

Reviewer #3: Yes

3. Have the authors made all data underlying the findings in their manuscript fully available?

The PLOS Data policy requires authors to make all data underlying the findings described in their manuscript fully available without restriction, with rare exception (please refer to the Data Availability Statement in the manuscript PDF file). The data should be provided as part of the manuscript or its supporting information, or deposited to a public repository. For example, in addition to summary statistics, the data points behind means, medians and variance measures should be available. If there are restrictions on publicly sharing data—e.g. participant privacy or use of data from a third party—those must be specified.

Reviewer #1: Yes

Reviewer #2: Yes

Reviewer #3: No

4.Is the manuscript presented in an intelligible fashion and written in standard English?

PLOS ONE does not copyedit accepted manuscripts, so the language in submitted articles must be clear, correct, and unambiguous. Any typographical or grammatical errors should be corrected at revision, so please note any specific errors here.

Reviewer #1: No

Reviewer #2: No

Reviewer #3: No

**5. Review Comments to the Author**

**Reviewer #1:** Manuscript need to be checked by a native English Speaker. for details, please also see the attached file.

Action: The necessary language corrections were reviewed and edited by Prof. Dr. Alen SOLDO who is a native English speaker.

species name must be italicized.

Action: The species names in the text were rechecked to be italicised.

what about topotyps and paratypes in discovered/renamed species?

Action: The specimen were collected at the locality at which the original type was normally distributed, so, they are all topotype. The locality of each speciemen was added in the Table to see topotype distribution of each species.

**Reviewer #2:**

Dear Authors, the work presented in this study is particularly interesting because it offers valuable tools for the identification of species within the Belonidae family, which are known to be very similar to each other. The genetic and morphological approaches used here are crucial for clarifying the taxonomic status of these species, particularly in regions like the Mediterranean and Black Seas where they are distributed. However, several aspects of the manuscript need to be addressed to improve its clarity and overall quality (please refer to the comments in the attached PDF for detailed feedback). Some sections of the paper appear to be unclear and repetitive, which could confuse readers and detract from the strength of your findings. Additionally, the English language used throughout the manuscript requires revision to enhance readability and ensure that the scientific content is communicated effectively. You also need to consider and discuss Ablennes hians. In summary, while the study contributes valuable insights into the taxonomy of Belonidae species, careful attention to the points mentioned above will greatly improve the manuscript’s impact.

Action:

- Necessary arrangements were made in line with the referee's suggestions in the main text.
- The vague and repetitive parts of the main text have been edited and streamlined for the reader.
- The entire text has been language checked by Prof. Dr. Alen SOLDO whose native language is English.
- Ablennes hians was used as an outgroup in the genetic part of our study. Since *A. hians* species is morphologically distinct from *Belone belone, Belone svetovidovi* and *Tylosorus imperialis* species, it was not evaluated in this section.

**Reviewer #3: Comments to the Redactor and Authors**

I thank the journal authorities and the Editor for handing me an opportunity to review a manuscript: “Systematic Identification of Needle Fish (Belonidae) Species using Molecular Genetic and Morphological Markers in the Mediterranean Sea”. Despite a number of serious concerns, I am confident that this research is competent. Nevertheless, I also suggest that the scientific significance of this manuscript is not appropriate for the journal PLOS ONE. In this manuscript authors try to clarify the taxonomic relationships between species within the genera Belone and Tylosaurus, using markers from the mitochondrial genome and morphological traits.

While the authors have used a significant number of specimens, the results they present are not of sufficient interest for a broad-ranging, multidisciplinary journal like PLOS ONE. Instead, I recommend that this work be transferred to a more specialized, thematic ichthyological journal that would be better suited to this manuscript.

**General comments:**

Authors need to significantly improve the quality of the manuscript, because nowadays it is not suitable for reading and understanding.

1. First of all, the manuscript is full of misleading typos, missing punctuation marks, and grammar mistakes.

Action: Spelling mistakes, missing punctuation marks and grammatical errors were meticulously corrected.

2. Second, the English in this manuscript should be significantly improved.

Action: The entire text has been language checked by Prof. Dr. Alen SOLDO whose native language is English.

3. Third, the quality of figures (maybe due to transfer to PDF) is low; it is impossible to read the legends on the figures.

Action: The original versions of the figures have already been uploaded to the system as a separate file.

4. Fourth, I did not find the NCBI numbers for COI and 12S rRNA sequences in the manuscript.

Action: The sequences used in the analyses were uploaded to the NCBI database and accession numbers were added to the draft text.

5. Fifth, today, and this is the rule, in taxonomic studies, it is necessary to use data not only from the mitochondrial genome but also from the nuclear genome. This is because the mitochondrial genome has predominant maternal inheritance.

Action: Although the use of nuclear DNA is increasing in taxonomic studies today, this does not mean that the use of nDNA is mandatory. In our study, 2 gene regions (COI and 12srRNA) from the mtDNA gene region were used. In addition, the genetic results of our study were supported by comparing with morphometric results. Therefore, there was no need to use nDNA in addition to the analyses used in the study.

Minor comments:

1. The manuscript title implies study of specimens from the Mediterranean Sea (not the Black Sea).

Action: In the title “Black Sea” is added as suggested.

2. Along the title and text: Please change "needle fish" to "needlefish".

Action: All “needle fish” corrected.

3. Gene names should be italicized in the manuscript.

Action: All gene region names in the text have been italicised.

4. Sub-species should be changed to subspecies in the text.

Action: All “Sub-species” corrected.

5. If a name of genera is mentioned once, then it should be abbreviated further in the text.

Action: Genus names are abbreviated as suggested.

6. It is necessary to add scales to Figures 9–11. The quality of Figures 4, 5, and 7–8 is poor, making it impossible to see anything in the phylogenetic trees. Figure 13 is unclear as to what it can show in evolutionary terms?

Action: Scale added to figures 9-10-11.

Action: Figures 4-5-7-8 uploaded to the journal system are of very good quality and clearly readable. Due to the large number of samples, the phylogenetic trees are too large to fit on one page.

6. PLOS authors have the option to publish the peer review history of their article (what does this mean?). If published, this will include your full peer review and any attached files.

If you choose “no”, your identity will remain anonymous but your review may still be made public.

Do you want your identity to be public for this peer review? For information about this choice, including consent withdrawal, please see our Privacy Policy.

Reviewer #1: No

Reviewer #2: Yes: Francesco Tiralongo

Reviewer #3: No
